# Communication of palliative care needs in discharge letters from hospice providers to primary care: a multisite sequential explanatory mixed methods study

**DOI:** 10.1186/s12904-022-01038-8

**Published:** 2022-09-06

**Authors:** Katharine Weetman, Jeremy Dale, Sarah J. Mitchell, Claire Ferguson, Anne M. Finucane, Peter Buckle, Elizabeth Arnold, Gemma Clarke, Despoina-Elvira Karakitsiou, Tracey McConnell, Nikhil Sanyal, Anna Schuberth, Georgia Tindle, Rachel Perry, Bhajneek Grewal, Katarzyna A. Patynowska, John I. MacArtney

**Affiliations:** 1 Interactive Studies Unit, Institute of Clinical Sciences, Birmingham Medical School, University of Birmingham, Birmingham, B15 2TT UK; 2 Unit of Academic Primary Care, Warwick Medical School, University of Warwick, Coventry, UK; 3 University of Sheffield, Sheffield, UK; 4 Marie Curie Hospice West Midlands, Solihull, West Midlands UK; 5 Marie Curie Hospice Edinburgh, Edinburgh, UK; 6 The University of Edinburgh School of Health in Social Science, Clinical Psychology, Edinburgh, UK; 7 Marie Curie Research Voices Group, Marie Curie, England, London, UK; 8 Marie Curie Hospice Bradford, Bradford, UK; 9 University of Leeds, Academic Unit of Palliative Care, Leeds, West Yorkshire UK; 10 Marie Curie Hospice Belfast, Belfast, UK; 11 Queen’s University Belfast School of Nursing and Midwifery, Belfast, UK; 12 Marie Curie Hospice Newcastle upon Tyne, Newcastle upon Tyne, UK

**Keywords:** Palliative care, Hospice care, Patient discharge summaries, Transitional care, Communication

## Abstract

**Background:**

The provision of palliative care is increasing, with many people dying in community-based settings. It is essential that communication is effective if and when patients transition from hospice to community palliative care. Past research has indicated that communication issues are prevalent during hospital discharges, but little is known about hospice discharges.

**Methods:**

An explanatory sequential mixed methods study consisting of a retrospective review of hospice discharge letters, followed by hospice focus groups, to explore patterns in communication of palliative care needs of discharged patients and describe why these patients were being discharged. Discharge letters were extracted for key content information using a standardised form. Letters were then examined for language patterns using a linguistic methodology termed corpus linguistics. Thematic analysis was used to analyse the focus group transcripts. Findings were triangulated to develop an explanatory understanding of discharge communication from hospice care.

**Results:**

We sampled 250 discharge letters from five UK hospices whereby patients had been discharged to primary care. Twenty-five staff took part in focus groups. The main reasons for discharge extracted from the letters were symptoms “managed/resolved” (75.2%), and/or the “patient wishes to die/for care at home” (37.2%). Most patients had some form of physical needs documented on the letters (98.4%) but spiritual needs were rarely documented (2.4%). Psychological/emotional needs and social needs were documented in 46.4 and 35.6% of letters respectively. There was sometimes ambiguity in “who” will be following up “what” in the discharge letters, and whether described patients’ needs were resolved or ongoing for managing in the community setting. The extent to which patients received a copy of their discharge letter varied. Focus groups conveyed a lack of consensus on what constitutes “complexity” and “complex pain”.

**Conclusions:**

The content and structure of discharge letters varied between hospices, although generally focused on physical needs. Our study provides insights into patterns associated with those discharged from hospice, and how policy and guidance in this area may be improved, such as greater consistency of sharing letters with patients. A patient-centred set of hospice-specific discharge letter principles could help improve future practice.

**Supplementary Information:**

The online version contains supplementary material available at 10.1186/s12904-022-01038-8.

## Introduction

### Background

Specialist palliative care aims to provide care and support for people with complex palliative care needs [1–3]. However, palliative care resources are not distributed equitably [4–6] with inequalities of provision associated with ethnicity, diagnosis, and age [7, 8]. Evidence [8, 9] suggests that referrals to palliative care are skewed towards white cancer patients although less is known as to whether such disparities manifest in discharge practices. Without reviewing and changing practices and policies, these disparities are likely to widen as the need for palliative care is increasing [10, 11], especially in community-based settings [9, 12, 13].

Co-ordinated and integrated [14], patient-centred palliative care, provided by primary and specialist palliative care within community and home settings are essential to a good death [2, 15]. Hospice specialist care supports those with complex palliative needs [3], but there is often differences between how primary care (GPs) and Specialist Palliative Care understand what palliative care needs are complex and what services might best support a patient [9, 16]. Efficient and effective cross-boundary communication is key, particularly as care transitions can risk patient safety [15, 17]. National [18] and international [19, 20] research indicates that communication issues are prevalent during care transitions [21], which can lead to adverse events such as readmissions [22, 23] and other consequences such as “inefficient use of [general practitioner] time” [24]. Little is known about the content of communication at the time of discharge from hospice-based specialist palliative care services into community and primary care. Furthermore, there is limited knowledge on what palliative care needs are associated with patients discharged from hospice-based care (e.g. physical, psychological, social and spiritual) or how these needs are communicated to general practitioners.

Hospices in the UK are a heterogeneous group of independently funded charities [25, 26] with government funding averaging 35% (range 20–50%) of hospices expenditure [27]. Hospices deliver a range of free-at-the-point-of-access generalist and specialist palliative services that can provide pain and symptom control, end-of-life care, and/or respite services. These can be provided by a multidisciplinary health and social care team at in-patient, out-patient, day service, or home locations [28]. Access to hospice services is ordinarily gained via referral from hospital specialists or General Practitioners (GP), and should include an assessment of holistic (physical, social, psychological and spiritual) palliative care needs [16].

A recent systematic review [7] by Wu & Volker, of live discharge from hospice in the United States, reported discharge rates from 5 to 23% [7]. In the United Kingdom, national guidance [29–31] suggests that discharge from a specialist service should include written communication that is sent to the clinician who will continue patient care; this is typically the general practitioner who acts as the family/primary care physician. Discharge communication is a complex practice in that the content, structure, and style may differ depending on local processes and the letter author [32–34]. Broadly, discharge letters should summarise “what has happened” (medication changes, treatment, tests and results …) and “what should happen next” (actions and plan for future care…) [30, 35]. Regulatory bodies recommend that general practitioners and patients should receive a (timely) copy of this letter [36–38]. However, primary care professionals report that hospital discharge letters frequently lack the information they require, which can adversely impact patient care and experience [35, 39, 40]. There is a limited evidence base on how these issues manifest in hospice discharge letters.

### Research question

What palliative care needs are described in hospice discharge letters, how is this communicated (and to whom), and why are these palliative care patients being discharged?

## Methods

### Aim

To explore patterns in hospice discharge letters, to identify what palliative care needs are associated with discharge and how palliative care needs are communicated in discharge letters (and to whom), and why these palliative patients are being discharged to primary care.

### Design

#### Overall study design

We used an explanatory sequential mixed methods design comprising retrospective review of discharge letters and focus groups with hospice staff. Mixing methods may offset the weaknesses and capitalise on the strengths of both [41–44], for example, quantitative data may be de-contextualised, but qualitative data can provide contextualised depth. We undertook an interpretative approach [44–46] to mixed methods of inquiry, which allows for the exploration of complex research questions [43] and in-depth understanding of the phenomena under scrutiny [42, 47].

#### Patient and public involvement (PPI)

A PPI representative (PB) who had lived experience of palliative care, in this case for his wife who died in their home shortly after discharge from acute care, was involved throughout the study. PB was involved in the study design, consulted on the study materials and findings, and attended advisory group meetings.

#### Stakeholder engagement

An advisory group met periodically throughout the project to discuss the study findings, and possible translation routes into policy and practice. This group comprised the research team, general practitioners, hospice clinicians, our patient and public involvement representative, and policy-makers. Our stakeholder engagement involving three UK hospices also established that discharge from hospice regularly occurs with live discharge rates (as % of admissions) between 24 and 35%.

#### Ethical considerations

Hospices were required to redact data before transfer to the research team. Focus group participants provided informed verbal consent to take part and were aware of their right to withdraw. Ethical approval was granted by University of Warwick Biomedical & Scientific Research Ethics Committee [ref. 154/20–21] on 28.07.2021. We also obtained local research approval by each hospice’s research governance committee.

### Setting

Data were from five hospices across the United Kingdom: three in England, one in Scotland, and one in Northern Ireland. Participating hospices had variable service organisation and provision but all offered inpatient services. Two hospices offered community nurse specialist services; all five had day-services; and four offered outpatient clinics.

### Retrospective case note review (quantitative data): description of materials

A sample of 250 discharge communications was deemed sufficient to allow descriptive analysis [9, 48] and was feasible based on preliminary information provided by hospices of the number of discharges that occur. It was likely to be large enough to quantitatively identify occurrences linked to discharge and also to undertake qualitative analyses that required reading of all letters. Discharge communication was defined as any electronic or hard copy form sent to the patient’s general practice, following inpatient/outpatient hospice care [32]. Such correspondence is often referred to as a “discharge letter”, and so we use this term. Each hospice selected 50 discharge letters from February 2020 (working consecutively backwards), in line with the criteria in Table 1. This pre-pandemic sample was to deliberately avoid discharges during COVID-19 pandemic peaks that were potentially skewed by the circumstances.

**Table 1:** Study inclusion and exclusion criteria

Inclusion criteria	• Patient documents that take a form of written discharge communication – this may include but is not necessarily limited to inpatient or outpatient discharge letters and discharge summaries. • Cases whereby the patient was discharged to primary care from one of the participating hospices. • Discharge communications between October 2019 and February 2020*. *If there are more than 50 letters within the sampling period, the 50 most recent should be selected, and if there are fewer, then the time period should be extended until 50 is reached.
Exclusion criteria	• Discharge documents to non-primary care services. • Documents that relate to patients under 18 years of age. • Documents relating to patients who have requested their records are **not** used for research.

Data from discharge letters were extracted by a clinician at each hospice using a standardised data extraction form adapted from Finucane et al. [9] (see Additional file 1). Patients’ needs were recorded as being included or not (Y/N) for physical needs, psychological needs, spiritual needs, social needs, functional care needs, and communication needs. Each data extractor also recorded reasons for discharge, diagnosis, date of referral, duration of care, and any advanced care planning. Patient case notes were reviewed by data extractors to collect supporting information including patient demographic characteristics (age, gender, ethnicity), and if and when the discharge letter was sent to the general practitioner and patient/carer.

To ensure consistency of coding, we provided training for data extractors and implemented the strategies recommended by Gilbert et al. [49] for case note reviews. Data extraction issues were discussed with the research team as they arose. Inter-rater reliability was assumed to be adequate where variant coding differences between hospices were +/− 10%, otherwise a 20% sample (10 letters) for the hospice was second-coded by KW. In cases of second-coder disagreement in the subset (K < 0.7), all letters for the outlier variant were re-coded by KW; this occurred twice.

### Focus groups (qualitative data): characteristics of participants

A local research fellow or clinician acted as collaborator for each hospice; they invited staff at their hospice to take part and facilitated the focus groups. Initial findings from the discharge letter review were presented to participants as part of a semi-structured approach to eliciting discussion on hospice discharge processes and communications (see Additional file 2 for focus group guide). Participants were asked to reflect upon their own experiences of discharging patients and contrast that to any patterns in the data presented. Focus groups were held virtually on Microsoft Teams and recorded. Audio was transcribed via Teams software (Stream) and checked by KW.

### Data analysis

#### Quantitative data

The data extracted from the forms were analysed descriptively using Microsoft Excel and SPSS, with variables compared and compiled across sites, to elucidate frequencies and trends. The discharge letters were also analysed using corpus linguistics methodology [50, 51] to identify communication and language patterns in terms of how palliative care needs are communicated in hospice discharge letters. Text from letters was imported to a software programme (*concordancer*) called *Antconc* [52] to build the discharge letter corpus. Language patterns of co-occurrence and relevance to the research question were identified with word frequency analysis whereby the concordancer calculates counts or “hits” (n) of words in the text samples or corpus. We focussed on lemmatised *content* words [53] (i.e. not functional or grammatical items such as “the”, “and”…), and then contextualised frequency results through concordance line reading [54, 55]. Lemmatisation [56] refers to consolidation of word variants including those marked for person or tense (e.g. patient(s)).

#### Qualitative data

Focus group transcripts were coded in NVivo and analysed following the six phases of a reflexive thematic approach (familiarisation; generating codes; constructing themes; revising and defining themes; writing-up) [57] by KW. Coding themes for the framework were discussed before coding commenced, informed by relevant literature reviews [33, 58], and refined through regular discussion between JM and KW.

### Triangulation of findings

Triangulation is a way to generate breadth of insights from multiple data sources [59–61]. Triangulated findings were developed iteratively and sequentially, as outputs from each method (descriptive statistics; corpus linguistics; themes) were produced. To do this KW and JM, with input from advisory group, used a constant comparison technique to compare and contrast findings within and across the quantitative and qualitative data sets to further elucidate patterns and differences.

We followed mixed methods guidance by O’Cathain et al. [62] and completed the “Good Reporting of a Mixed Methods Study” (GRAMMS) checklist (Additional file 3).

## Results

### Retrospective case note review

Discharge letters for 250 patients were examined (54% male; 46% female). Of these, 248 (99.2%) were inpatient discharges and two were day therapy discharges (0.8%); 249 (99.6%) were medical physician discharge letters (typically sent between doctors), and one was a nursing discharge letter (typically sent between nursing staff). Most discharged patients were aged between 50 and 89 years (*n* = 220, 88%) and of white ethnicity (*n* = 226, 90.4%), with a range of 80–100% across hospices (see Additional file 4). The total number of words in the discharge letter corpus was 90,598 with 4048 different word types. The 100 most frequent content words (i.e. nouns, verbs…) for the corpus are in Additional file 5 alongside concordance samples; the top 25 content words are in Table 2.

**Table 2:** The 25 most frequent content words in the discharge letter corpus (lemmatised)

Rank	Hits	Content word	Word form(s)
1	970	patient	patient 967 patients 3
2	857	care	care 831 cared 16 cares 2 caring 8
3	774	home	home 770 homes 4
4	712	pain	pain 705 pains 7
5	688	admission	admission 664 admissions 24
6	546	place	place 538 placed 6 places 2
7	534	hospice	hospice 534
8	508	discharge	discharge 386 discharged 117 discharges 2 discharging 3
9	451	prefer	prefer 15 preferred 428 preferring 1 prefers 7
10	421	during	during 421
11	383	plan	plan 106 planned 23 planning 97 plans 157
12	338	follow	follow 183 followed 35 following 119 follows 1
13	331	day	day 241 days 90
14	274	problem	problem 15 problems 259
15	274	symptom	symptom 129 symptoms 145
16	267	feel	feel 50 feeling 31 feelings 4 feels 51 felt 131
17	267	time	time 168 times 98 timing 1
18	261	team	team 252 teams 9
19	250	dose	dose 206 doses 43 dosing 1
20	242	community	community 242
21	242	due	due 242
22	242	use	use 84 used 48 uses 37 using 73
23	228	family	families 1 family 227
24	227	increase	increase 51 increased 123 increases 2 increasing 51
25	226	death	death 226

**Fig. 1:**
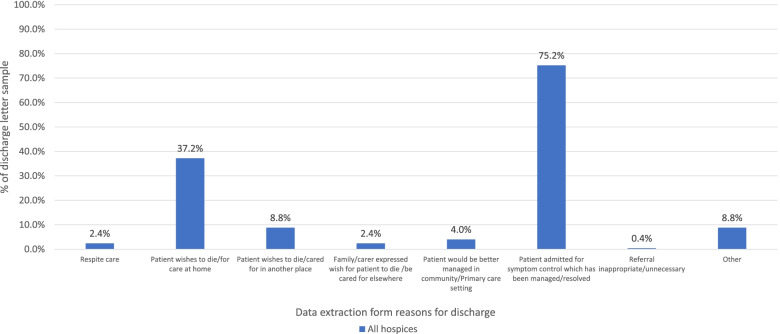
Documentation of reasons for discharge in discharge letters

Most patients had a diagnosis of cancer (*n* = 205, 82.0%); for 199 of these patients, cancer was the primary diagnosis (79.6%) with a range of 70–96% across sites. 45.6% of patients (*n* = 114) had co-morbidities documented in the discharge letters. The documented reason for admission for most was symptom management (*n* = 232, 92.8%). Admission length ranged from 1 to 240 days (median 17 days). The main reasons for discharge given were symptoms “managed/resolved” (*n* = 188, 75.2%), and/or that the “patient wishes to die/for care at home” (*n* = 93, 37.2%) (see Fig. 1 for reasons for discharge results).

**Fig. 2:**
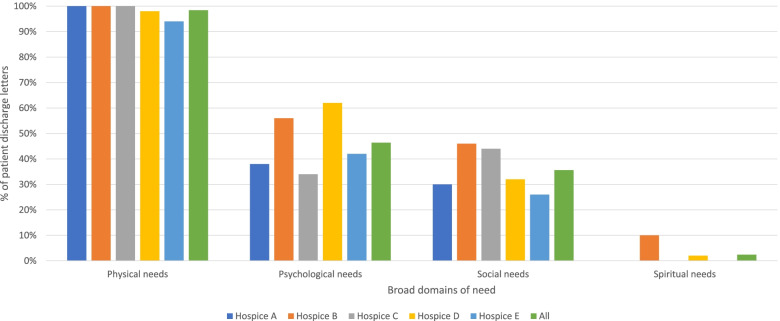
Broad domains of need in hospice discharge letters

Patients’ needs described in discharge letters included those that led to admission, those managed during the admission, and those which were ongoing at the point of discharge. Documented needs covered physical, social, psychological, and spiritual domains (see Fig. 2); this included 84 (33.6%) with one domain, 115 (46.0%) with two, 45 (18.0%) with three, and two (0.8%) with all four.

Four patients (1.6%) had needs separate from the domains, described as functional needs for two, with the other two admitted for respite care. Other patient needs included: “capacity or communication needs” (*n* = 27, 10.8%), “functional needs” (*n* = 124, 49.6%), and “changing/dynamic needs” (*n* = 126, 50.4%) such as change in condition (*n* = 86). Carer/family needs were coded for 31 letters (12.4%). Additionally, the patient advanced care plan (ACP) was noted as discussed or updated in 222 discharge letters (*n* = 88.8%). Further data extraction results are in Additional file 6.

### Focus groups

Five focus groups took place, one for each participating hospice, in November 2021. Groups took around 1 h; recordings ranged from 52 to 59 minutes. In total, 25 participants took part with 3–6 per site, including doctors (*n* = 15), nurses (*n* = 4), and allied healthcare professionals (*n* = 6). Six themes were identified; these are listed in Table 3.

**Table 3:** Themes identified during analysis

Code	Illustrative participant quotation
Structure and content of discharge letters	*“I did use the format that [CLINICIAN] suggested, which was basically diagnosis, problems like the issues, what do you want [GP] to do about it… literally diagnosis, what the issues were, what the actions were, and if I wanted to chat about all the other stuff, just to put that underneath, and if we ever need it, it’s good to have it there.”* [PCS9]
Communicating patient (complex) palliative needs	*“the GP Uh, needs to know that this pain was complex and challenging, and [if] it’s not written down. Then there is a fail.”* [PCS18]
Responsibility of care	*“I think in our heads we’re quite clear about responsibility, but maybe we’re not. But I always think, you know, when someone is in the building they’re our responsibility, when they leave the building, we have responsibilities to them to provide specialist palliative care, but the ultimate responsibility is with the primary care.”* [PCS11]
Patients receiving letters	*“I was actually surprised that we don’t, there was one letter that was copied to a patient, but other than that we don’t routinely copy letters and it did make me think actually, should we be copying, you know, giving copies of letters to patients.”* [PCS23]
Variability in mode and transmission of discharge communication	*“I think members of the team maybe do things slightly differently as well. Sometimes it might be an email to the GP. Sometimes it might be the more formal letter, the GP letter from the nursing team, or sometimes it is, it’s just telephone call correspondence...”* [PCS13]
Computer systems, shared and cross-service integrated records, and technology issues	*“I would say the challenge is that everybody you know GPs are on a different computer system to us who are separate from the hospital so that you know the hospital can’t see our discharge letters...”* [PCS12]

### Triangulated findings

#### Structure, content, and transmission of discharge letters

Discharge letters varied in format from structured or templated letters to free text or narrative-style letters, as well as combinations of both. The mode of sharing discharge letters varied, with references to electronic and hard copies of letters as well as synchronous and asynchronous communication (see Table 4). In many cases (*n* = 158, 63.2%), the letter was sent to the general practitioner on the day of discharge (*n* = 113) or < 48 hours (*n* = 45); there were no cases where this exceeded 3 weeks. However, some participants did identify timing of letters as an area for improvement:

*“I think what we can improve on is how quickly we send the letters, ‘cause I think especially for medicines it’s a slow turn around from a [general practitioner]. And to getting it from your pharmacy and things like Medi boxes, we don’t send them straightaway.”* [PCS4].

**Table 4:** Variability in transmission of discharge communication

Modes and formats used	Quotation exemplar from hospice focus groups
Electronic and hard copies of medical discharge letters	*“I think now they’re only printing two copies, so for the patient [and] for the district nurse, the [general practitioner] one either gets sent virtually yeah, electronically”* [PCS10].
Phone calls	*“If there’s stuff I really want [the general practitioner] to do for a patient that is discharged at, I ask them like I phone them.”* [PCS24]
Email	*“I think they [LETTER] will go by NHS email.”* [PCS10]

The content and depth of detail in the letters varied across the sample, both within and between hospices, something that was acknowledged by focus group participants. For example, several participants acknowledged that letters were often idiosyncratic and dependent upon clinician preferences and experience:

*“I think every medic has their own style essentially I am very wordy person. I would write probably 3 pages letter and it would probably include psychological distress. Other people may not add that. So I think depends on how experienced you are… It’s a personal style of writing the letters.”* [PCS17].

*“We come with our own sort of prejudice and bias in what we think is important…if you have a particular interest in sort of the psychology aspects of things then you’re more likely to like add that in...”* [PCS7].

One participant outlined the function of the discharge letters and their purpose in a broader sense, *“This is meant to be a holistic letter that describes a person so that the receiving [general practitioner] or whoever reads it get a full picture of this persons’ needs”* [PCS18].

Despite variation, there were some commonalities in letter content; for example, the patient’s diagnosis was extracted for all letters, and corpus linguistics analysis found the following content elements occurred in over 100 letters: “care” (244 letters); “plan(s)” (178 letters); “follow up” (130 letters); and “management” (105 letters). “Care” frequently co-occurred or *collocated* [63, 64] with “preferred” (log-likelihood = 4221.6) and “place” (log-likelihood = 3821.7); see concordance line sample in Table 5.

**Table 5:** Sample of 10 random concordance lines for “prefer”

breathing techniques and hand-held fans and	**prefers**	a cool breeze from an open window.
on this in the past with good effect.	**preferred**	place of care: home preferred place of death:
during hospice admission: Not for resuscitation	**preferred**	Place of Care: Preferred place of care – home
discussions. Details of advance care plans	**preferred**	place of care - home Preferred place of death:
at the hospice. Details of advance care plans	**preferred**	place of care - home Preferred place of death:
and dying. Preferred place of care: Home	**preferred**	Place of Death: Home- but doesn’t want
Place of Care: Preferred place of care - home	**preferred**	Place of Death: Preferred place of death:
of care - home Preferred Place of Death:	**preferred**	place of death: home. Deciding Right
DNAR in place and is in agreement. Her	**preferred**	places for care and death are home. If
to be less well at home, then her	**preference**	would be to be admitted to [PLACE]

A recurring pattern was “preferred place of care/death” for the patient. Similar constructions to “death” were found for “die/dying” with references to preferred location of death and patient/family knowledge of terminal nature of disease (*“she knows she will die from this”*) in addition to any issues or barriers to advanced care planning e.g. fear (*“fear of dying/being a burden and very anxious which makes all advance care planning discussions difficult”*).

The reasons for discharge were not always clear in the letters, and sometimes required interpretation by the clinician data extractors. As one extractor said,

*“the reason for discharges was really commonly not specified and…there are quite a few times that I was left thinking I don’t know whether their symptoms have really been sort of managed um, but they’ve been discharged.”* [PCS23].

Ways to improve content and quality of discharge letters included, for example, enhanced training for junior doctors and checking of letters by senior clinicians, *“I think junior doctors need guidance on how to write discharge letters and what the discharge letter is… there’s not a formal process saying that a senior doctor needs to check it…”* [PCS25].

Several focus group participants summarised what discharge letters elements they thought were important or necessary for a *“good discharge letter”,* for example:

*“So diagnosis, reason for admission, what we’ve done to them while they have been here, what their future holds so advanced care planning, preferred choices, what their status is and any social concerns maybe”* [PCS1].

However, some participants felt letters are generally comprehensive and content improvement should focus on *how* to highlight pertinent and urgent information to general practitioners, such as use of bold font and/or headlining key actions and information:

*“I’ll do like the meat bulk plan and then action, yeah for [general practitioner] and I put that in bold so if it’s describing something so that they may not read all of that, but at least they’ll have a look.”* [PCS3].

Overall, we found that there was a range of ways used to communicate discharge information, and that there did not appear to be a consensus on the structure and content of letters that is most helpful for primary care teams.

#### Communicating patient (complex) palliative care needs

Specialist palliative care seeks to provide holistic care to patients. Nevertheless, there was a marked focus on physical needs (*n* = 246, 98.4%) in the discharge letters (see Fig. 2), with pain the most frequent (*n* = 194), followed by constipation (*n* = 87), breathlessness/secretions (*n* = 79), fatigue (*n* = 61), and nausea/vomiting (*n* = 54). The second most documented domain of need was psychological/emotional needs (*n* = 116, 46.4%) such as anxiety (*n* = 62), depression (*n* = 26), grief (n = 8), and anger (n = 7). Social needs were the third most documented (*n* = 89, 35.6%), for example, housing (*n* = 20) and social isolation (*n* = 11).

Spiritual needs were the least documented domain of need; this was indicated both in data extraction results (*n* = 6, 2.4%) and corpus results – “spiritual” had only one hit and the following terms did not occur in any letters: “pastoral”, “religion”, “meaning of life/death”, “existential”. Reasons for low recording of spiritual needs in letters were described by focus group participants as patient and clinician preferences, it was not thought relevant, and perceptions that spiritual needs were too personal to include in the letter. Issues with confidence and understanding were also raised: *“It doesn’t surprise me that we don’t reference spiritual needs that that much…I think it’s an area we recognize we’re not great at addressing in person, let alone writing.”* [PCS7].

Reasons for discharge included recognition that some discharges occur because referral has taken place *too early* before complex palliative care needs had developed that necessitated intervention:

*“I think the commonest reason for discharge there is, so discharging them from the whole service, is because the referral has come in very early in the patients illness and actually they don’t have any specialist palliative care needs…there’s been a misunderstanding by the referrer… as to what our role is and so that the kind of traditionally held role that you know we are…[going to] provide emotional support to the patient. Actually now we discharge people that just need that because that’s not considered a specialist palliative care need.”* [PCS25].

Several participants suggested that all hospice patients should have an element of complexity in their palliative care needs, and yet participants found “complexity” was *“…really hard to define… it might be a number of variable factors that make it complex…”* [PCS8]. In order to directly acknowledge when “complex(ity)” applies, some participants outlined that letters should use more explicit and clear language:

*“They probably did have complex pain, and it’s maybe our annotation of that. It makes it look as though their pain wasn’t a complex symptom, and I believe our discharge letters are freestyle. It’s a narrative, as opposed to ticking complex pain or whatever, so it’s a learning point for us to not underestimate or not describe things as less than they are.”* [PCS18].

Variability in use of “complexity” was reflected in the discharge letters. The corpus linguistic analysis found that there were only 16 hits of “complex(ity)” across 14 letters (see Table 6). As seen in Table 6, there were differing contexts for the word “complex(ity)” in the letters. These included references to complex needs and complex medication as well as symptoms.

**Table 6:** All concordance lines for “complex(ity) in hospice discharge letter corpus

and upper thoracic spinal metastases,	**complex**	and difficult to assess mixed somatic and
of discharge home or to hospital based	**complex**	care and he chose to go home. [PATIENT]
home placement. He has significant and	**complex**	care needs including non-invasive ventilation
care home and of hospital-based continuing	**complex**	care were both discussed but [PATIENT]
Hospice – currently twice a week due to	**complexity**	. Community Palliative Care Team: Future
admitted for symptom control. Presents with	**complex**	neuropathic pain secondary to local invasion
his sister’s but in view of the	**complexity**	of his medicines and previous anxiety
I have advised [PATIENT] that given the	**complexity**	of issues related to his symptoms, that
to the hospice from PLACE for: 1.	**Complex**	pain control- Neuropathic pain in left arm
anatomy of his metastatic cancer he has	**complex**	pain. His pain is in his upper back
was admitted to the [HOSPICE] for	**complex**	pain management and low mood. [PATIENT]
problems during this admission: 1. Pain:	**complex**	pain management. [PATIENT] is focused
treatments, interventions [PATIENT] has	**complex**	pain which is long standing and difficult to
cream. 5. Pain — from recent fall, OA,	**complex**	regional pain syndrome. We tried [PATIENT]
be eligible for fast-track funding due to	**complex**	symptoms and recent deterioration. This
home today [DATE] and due to his	**complexity**	we decided as a team to hold his

The most frequent content collocate for “complex(ity)” was “pain”. In data extraction forms, documentation of complex pain varied; pain (*n* = 194) was coded by data extractors in 77.6% of discharge letters with a range of 74–86% across hospices. “Complex pain” was coded for 74 patients (29.6%) with a range of 18–66%. For 73 of these, one or more of the complex pain markers (e.g. drug sensitivities; see Additional file 1) had been coded, although combinations of markers varied. This lack of agreement on what constitutes complex pain was reiterated through the differing viewpoints expressed in focus groups, with some participants suggesting it is a *“total pain”* or involves a psychological element, and others stating that complex pain is that which cannot be managed in the community and therefore is “*whatever pain brings you to the hospice*.” Notably, the latter view was not reflected in data extraction results as “complex pain” coding was lower than that of “pain” for all hospices. A few hospice staff members suggested that “complex pain” is context-dependent, *“because…of the situation”* [PCS12], or influenced by the perception of the treating clinician:

*“…the person came in with pain and we threw everything at them and we still couldn’t manage it. That I think that to us would be complex pain, whereas to a [general practitioner] it’s maybe they’re on four or five medications. I mean, all the patients we admit with pain are on that, so I suppose it’s your context of complex pain.”* [PCS24].

Overall, the results suggested a lack of consensus on what constitutes “complexity”.

#### Responsibility for future and ongoing care

A sample of lines for the word “discharge” from the sample of discharge letters is found in Table 7 below.

**Table 7:** Sample of 10 random concordance lines for “discharge”

need to be reviewed in the community after	**discharge**	2. Blood transfusion of two units was done as
has her own health problems and was only	**discharged**	from hospital on DATE. Dad is AGE old.
pain, preferring to use paracetamol instead. At	**discharge**	her pain is well managed. Peripheral oedema
oral morphine when required. She is being	**discharged**	home with clinical nurse specialist follow up
Physical and cognitive function on	**discharge**	No change in cognitive function. Physically
and ordered equipment felt helpful for his	**discharge**	PATIENT aware that he has lung cancer and
or constipation during his time at [PLACE].	**Discharge**	plan: Equipment required: Riser recliner to be
to a Psychology referral being completed on	**discharge**	Plans for future care: Will be reviewed at
was not in place at the point of	**discharge**	The [PLACE] FastTrack Service will
Equipment required: home visit being done on	**discharge**	today Care package: awaited but didn’t want

Differing perspectives were expressed in the focus groups in relation to what “discharge” is, and this was associated with ambiguity in what future care in the community entailed. Terms surrounding community care provision were not frequent in the corpus (e.g. nil hits of “primary care” and “[general practitioner]/GP” occurred in 28 letters); the exception was the word “community” itself (*n* = 242 in 154 letters). Instances of “community” were typically either statements that the patient had been discharged to the community, or relating to management plans and instructions for the community team, e.g. *“This will need to be monitored in the community.”* Such language does not necessarily make it clear whether follow-up was to be undertaken by the hospice community palliative team, the primary care team, or both. In the focus groups, some participants posited that primary care undertake this, e.g. “…*patients now will be receiving support from [general practitioners]” [PCS25]* and others suggested differently, *“although they’re being discharged, they’re not…I’m imagining it’s vanishingly rare that we ever discharge to the [general practitioner]”* [PCS7]. One participant summarised: *“I just feel like there may be that disconnection with within the community, but it’s hard to know because we are not out there.”* [PCS20].

In summary, there was a diversity of views and a degree of ambiguity on who was responsible for the patient upon discharge.

#### Sharing discharge letters with patients and carers

Data extractors could only code whether the discharge letters had been shared with the patient or carer if this was explicitly noted in the letter itself, or recorded in the patient’s notes. Documentation review found that verbal information had been provided to the patient/carer in 130 cases (52%), and that discharge letters had been provided to patients in 52 cases (20.8%) with one instance of the carer receiving the letter (0.4%). Of these 53 instances of patient/carer letter receipt, there were only 3 letters which were identified as explicitly noting this. Corpus searching found only one hit of copy(ies/d), which supports the finding that most letters do not record that a copy was provided to the patient/carer. In all cases where patients/carers were identified as having received the letter, this took place on the day of discharge.

Several focus group participants (for three hospices) noted that letters are not usually sent to patients, e.g., *“none of the 50 did [get sent to the patient] and I spoke to our admin about that, and they said that they would only send one if we asked for one.”* [PCS2]. Another focus group participant explained that patients receiving discharge letters is not reliably undertaken, *“I’m sure a while ago we were told to offer them. But then I think that fell by the wayside”* [PCS24]. Additionally, one focus group raised consistency issues with recording of sending letters to patients, “*we’re not necessarily good at capturing that”* [PCS7].

There appears to be inconsistency within and across hospices, both in providing patients with discharge letters and in documenting what has been provided. During focus groups we explored why this might be. Some participants expressed concerns or outlined difficulties in writing a letter that *“covers everybody’s’ needs”,* particularly relating to aspects that could be perceived as sensitive or distressing for patients, such as future care planning:

*“…say for example, patients haven’t wanted to talk about some end of life issues and then you’ve got to say you have got to sort of communicate that to the community team. But how do you communicate that then without upsetting the patient, if they’ve not wanted to talk about it when they go home?”* [PCS24].

Several participants commented on the benefits of patients and carers receiving letters, as well as suggesting that they support this practice:

*“…you can speak to people about certain things and it’s like they’ve forgotten or they’ve you know they’ve not really taken it on board and maybe if it was in writing and they had it in the letter then at least they can read over it and see you know what happened”* [PCS2].

*“I will say the value of a discharge letter for a patient is that they have something, they have a physical copy in their hands such that where communication may be delayed or say - heaven forbid – they get discharged from the hospice and end up in hospital that night. They’ve got an active, up-to-date record and actually a lot of patients feel very empowered with those letters.”* [PCS8].

For 48 patients, they received a personalised discharge letter instead of a general practitioner copy; this was due to convention at one of the hospices. This was discussed at the focus groups with suggestions that the language of such letters has to be different or that the content should be abridged, *“…not like a detailed letter, that we would send to primary care”* [PCS11]. There were mixed views on the concept of patient personalised letters with some participants not supporting this practice within the context of hospice care, *“[Ours are] not written like that and I think it’s right that we don’t write them like that”* [PCS10].

We found that there was no consensus on whether discharge letters *should* be shared with patients or carers, nor – if they were shared – what format this might take.

## Discussion

### Main findings/results of the study

Our first study objective addressed the question, ‘what palliative care needs are associated with hospice discharge?’ Our examination of 250 discharge letters from five UK hospices found that physical needs were referenced in nearly all discharge letters (98.4%) and most patients were admitted for symptom management (92.8%). This suggests a focus on management of physical needs, particularly that of pain, which was the most documented. The proportion of discharged patients with managed symptoms was noticeably lower (75.2%) than those admitted for symptom management (92.8%); this could suggest insufficient information in the letter itself and/or that patients are being discharged with unresolved or ongoing symptoms which may be due to other reasons for discharge (e.g. patient wishes to die or be cared for at home).

The second study objective focussed on how palliative care needs are communicated in discharge letters (and to whom). We found that there was marked heterogeneity in the structure and content of discharge letters sent to general practitioners from the five hospices studied. Comparably to referral letters to palliative care [9], participants acknowledged that quality and content of discharge letters may be influenced by writer preferences and experience, as well as available resources. It was not routine practice at all participating hospices for patients to receive discharge letters or for this to be recorded. When they did, the format varied and could include a patient-directed/personalised letter, a nursing letter, or a copy of the medical letter to the general practitioner. Patients not receiving hospice discharge letters may represent a missed opportunity to involve patients in their care, including signposting them to community-based support, both in-hours and out-of-hours.

The third and final study objective looked at why palliative patients are being discharged to primary care. The main reasons for discharge given were that the patient’s symptoms had been managed or resolved (*n* = 188, 75.2%) and that that the patient wished to receive care or die at home (*n* = 93, 37.2%).

### Strengths and weaknesses/limitations of the study

This study highlights variation in hospice discharge communication practices. The study was limited to 250 discharges, but we purposefully included hospices from across the United Kingdom to increase the applicability of findings. Our data collection could only record patients receiving letters where it had been documented either in the letter itself or the patient notes. It is probable that a higher number may have been received in practice, but without documentation of this process it cannot be determined how many. Thus, our results should be interpreted cautiously and are not a depiction of prevalence.

We reduced measurement bias by having clinician data extractors who were separate to the team undertaking analysis [9]. However, due to resource constraints there was only one extractor per site and it was not possible to blind them to the aims of the study. Interpretation of data and analyses can be influenced by the identities and attitudes of the researchers [65, 66]. Therefore, to help account for subjectivity, reflexivity [65, 66] was practised through KW making notes during the study for reflection upon researcher interpretations, as well as regular discussions on ideas and preconceptions between JM and KW, and stakeholder meetings with the wider team. Such reflexivity may be seen to reduce but not eradicate bias [65, 66].

This study mainly focussed on medical discharge letters sent from discharging physicians to the patient’s General Practitioner (99.6%). Other types of hospice discharge letters are sent between teams, for example, social workers, but these were not captured as our study examined those letters typically sent to the patient’s general practice. The study sample primarily relates to inpatient discharges (99.2%). This was due to difficulties running reports to search for community discharges, combined with the rarity of these discharges. Several hospices advised that discharges from *all* hospice services seldom occur; from our data we cannot determine the prevalence of this nor the implications.

### What this study adds and implications of the findings

We found that those discharged are predominantly white, aged between 50 and 89, with a diagnosis of cancer. Comparison with a study exploring referral to hospice [9] finds patient characteristics of the two populations (referred and discharged) are similar. This suggests that the causes of inequities in care may lie further upstream, in understanding of what specialist palliative care is and who it is for.

The broad pattern of the four domains of palliative care needs [16, 67] appeared to mirror that of referral documentation in a study by Finucane et al. [9] in that physical needs were the most documented domain followed by psychological needs, social needs, and spiritual needs. As previous studies have demonstrated, there is meaningful uncertainty both within primary care [16] and specialist palliative care [9] about patient suitability for referral. Our findings support these studies [9, 16] first, by highlighting ambiguity in communicating assessments of complex needs and pain, with several participants recognising that “complexity” is difficult to objectively define [3, 9, 16]. For example, definitions of “complexity” [15, 16, 67] in palliative care often include reference to “spirituality” but spiritual needs were rarely referenced in the data (2.4%). Secondly, some participants explained how referred patients should have an element of complexity in their needs, and that one of the reasons for discharge outlined in focus groups was that the referral had taken place too early in the disease trajectory i.e. before the patients’ needs had become complex. Although our study  was unable to show the extent to which this happens this reasoning contradicts those research findings and initiatives [1, 68–70] that encourage early referral to help reduce or prevent development of complex needs [9].

Finally, we found that letters rarely directed general practitioners to key indicators of complex needs or pain, which could help develop a better shared understanding of what constitutes these. As discharge letters are expected to communicate “what has happened” and “what should happen next” [30, 35], we expected to see descriptions of changing (complex) palliative care needs [3], advice on management of ongoing needs, along with who would be responsible for providing that care. However, there was sometimes ambiguity in “who” will be following up “what” in the discharge letters, and whether described patients’ needs were resolved or requiring further management in the community. This is important as many healthcare systems around the world prepare for increased demand in palliative care, we can expect specialist palliative care to assess and discharge more patients back to the community. If communication between the two services is unclear or poor, patient care is likely to suffer.

Our study indicated a number of ways in which discharge letters can be improved. “Reason(s) for discharge” and “plan(s)” should always be included in letters as well as an indication of whether patients’ needs are those that led to admission, those addressed or resolved during admission, or ongoing needs which require further management and support. Discharge letters should also clearly indicate any actions for primary care, and specify if and how the specialist palliative care service may be continuing to support the patient and family in the community setting; this would provide increased clarity as to who is providing what service and support, to ensure patients’ and families’ needs are met and actions are not missed. Alongside this, we recommend review of discharge processes to explore the possibility of widening multidisciplinary team access to the discharge letter. We suggest that discharging physicians should carefully consider the relevance of each domain of need (physical, psychological, social, and spiritual) as well as any changes or updates to the patient’s ACP; these could be mapped across to the discharge letter, as relevant, to help structure narrative text and better ensure that non-physical needs are not over-looked in the care plan or discharge letter itself, thus providing a more holistic picture of patient and family need.

Findings of this study indicate that patient receipt of discharge letters and recording of this is inconsistent in practice. This is despite patients receiving discharge letters being endorsed by a range of guidance, initiatives, and past research [33, 37, 38, 71–73]. As a result of this inconsistency, patients may lack awareness of the information that has been communicated to their general practice team and of any care advice that has been given. Our previous realist review [33] and realist evaluation [34] on hospital discharge letters found that patient preference for receiving their discharge letters is generally high and that there are many benefits of this practice, including increased patient satisfaction and sense of involvement [72, 74]. Future research needs to consider whether these findings occur within hospice discharge contexts, particularly accounting for experiences of general practitioners, patients, and carers. This should consider the benefits of hospice-specific discharge letter principles to aid standardisation of quality of communication and improve consistency of letter content, comparable to that currently available to hospitals [29, 30, 75, 76].

## Conclusion

Our study has provided insights into hospice discharge communication. We found that the content and structure of discharge letters was highly variable, and that they mainly tended to focus on physical needs. The responsibility of care and actions (i.e. *who* should do *what*) needs to be more explicit in discharge letters. Our findings suggest that patients are not consistently receiving discharge letters. While the impact of this remains unknown, it is likely that this will be contributing to gaps in the coordination and quality of care, and a lack of continuity between hospice and community-based services, at a time when the patient is most in need of patient-centred care.

## Supplementary Information

**Additional file 1.** Data extraction form.

**Additional file 2.** Focus group guide.

**Additional file 3.** GRAMMS checklist.

**Additional file 4.** Aggregated special category data.

**Additional file 5.** Corpus linguistics outputs.

**Additional file 6.** Data extraction results.

## Data Availability

All data which can be made available are included in the article or uploaded as supplementary information. Despite names and other identifiers being removed, the in-depth nature of discharge letters and the focus group data may mean that participants could be identified. Therefore, quotes and extracts have been used but these data in full form are not publicly available. To request the data from this study, please contact author Dr. John MacArtney.

## Abbreviations

ACP: Advanced Care Plan
GP: General Practitioner
OA: Osteoarthritis
UK: United Kingdom
