# Supplementary material for: Communication of palliative care needs in discharge letters from hospice providers to primary care: a multisite sequential explanatory mixed methods study
Source: BMC Palliat Care. 2022 Sep 6;21:155. doi: 10.1186/s12904-022-01038-8 (PMC9444706; doi:10.1186/s12904-022-01038-8)
Supplement: Supplementary file 1 — Additional file 1. Data extraction form. [file 12904_2022_1038_MOESM1_ESM.docx]

**Additional file 1 Data extraction form**

**DISCHARGE FROM HOSPICES STUDY**

**Data extraction template for part 1 (discharge letter review)**

Please complete this form* for each of the discharge letters selected for the study by your participating site.

**PLEASE COMPLETE -**

**Hospice site: ___________________________________________________**

**Patient date of referral to specialist palliative care (if known): ___________________**

**Reason for referral: ___________________________________________**

**Date of referral acceptance (if known): _____________________________________**

**Patient date of discharge from specialist palliative care: _______________**

**Patient primary diagnosis: ________________________________________**

| **Discharge letter review** | | Tick (✓)  all that apply |
| --- | --- | --- |
| **Domain or markers of complex needs** | **Individual marker / variant** |  |
| **1. Physical Symptoms** | 1.1 Physical Symptoms ANY |  |
|  | 1.2 Pain ANY |  |
|  | 1.3 Complex pain |  |
|  | 1.4 Syringe driver |  |
|  | 1.5 Two or more types of analgesia or routes of administration prescribed |  |
|  | 1.6 Pain required IPU admission |  |
|  | 1.7 pain requiring high dose opiates” (>300mg /24hrs orally or 100m /24hrs) |  |
|  | 1.8 Any of the following prescribed: Methadone, Alfentanil, Clonazepam, Lidocaine plasters, Abstral, Nifedipine |  |
|  | - 1. Ketamine administered/prescribed |  |
|  | 1.10 Pain without physiological explanation/ pain with psychological element |  |
|  | 1.11 Psychology referral made for pain management |  |
|  | 1.12 Drug sensitivities or multiple drug allergies that limit options |  |
|  | 1.13 Patient has history of /concurrent drug abuse |  |
|  | 1.14 Family members or person in home setting has history of drug abuse |  |
|  | 1.15 Patient requires interventional anesthetics |  |
|  | 1.16 Anticipatory prescribing/ just in case medicines |  |
|  | 1.17 Breathlessness / shortness of breath/ Secretions |  |
|  | 1.18 Nausea/ vomiting |  |
|  | 1.19 Confusion |  |
|  | 1.20 Agitation |  |
|  | 1.21 Constipation |  |
|  | 1.22 Fatigue |  |
|  | 1.23 Other (state what) |  |
| **2. Psychological, Emotional or Spiritual Needs** | 2.1 Psychological / Emotional Need ANY |  |
|  | 2.2 Psychological/emotional needs of patient relating to anxiety |  |
|  | 2.3 Psychological/emotional needs of patient relating to depression |  |
|  | 2.4 Psychological/emotional needs of patient relating to anger |  |
|  | 2.5 Psychological/emotional needs of patient relating to avoidance |  |
|  | 2.6 Psychological/emotional needs of patient relating to collusion |  |
|  | 2.7 Psychological/emotional needs of patient specifically relating to grief |  |
|  | 2.8 Psychological/emotional needs of carer specifically relating to grief |  |
|  | 2.9 Psychological/emotional needs of family/friends specifically relating to grief |  |
|  | 2.10 Psychological/ Emotional Need of carer ANY (if relevant) |  |
|  | 2.11 Psychological/ Emotional Need of family/friends ANY (e.g., depression) |  |
|  | 2.12 Spiritual needs of patient |  |
|  | 2.13 Spiritual needs of carer (if relevant) |  |
|  | 2.14 Spiritual needs of family/friends |  |
| **3. Diagnosis & Active Co-morbidities** | 3.1 Active co-morbidities ANY [if YES please list below]  [TEXT HERE]  [TEXT HERE]  [TEXT HERE]  [TEXT HERE] |  |
|  | 3.2 Patient diagnosed with cancer |  |
|  | 3.3 Cancer is primary diagnosis |  |
| **4. Functional Care Needs** | 4.1 Functional care needs ANY  [If YES, please list below e.g. problems with shower at home]  [TEXT HERE]  [TEXT HERE]  [TEXT HERE] |  |
| **5. Social Situation** | 5.1 Social situation ANY |  |
|  | 5.2 Social exclusion or isolation |  |
|  | 5.3 Social responsibilities |  |
|  | 5.4 Housing |  |
|  | 5.5 Other social factor (state what) |  |
| **6. Capacity or Communication Needs of Patient** | 6.1 Capacity / Communication needs ANY |  |
|  | 6.2 Capacity needs |  |
|  | 6.3 Communication needs (e.g. deaf/hearing loss) |  |
| **7. Patient Characteristics and identity** | 7.1 Patients coping mechanisms/behaviours ANY |  |
|  | 7.2 Patient ethnicity or race belonging to ethnic minority group (Asian, Black, African, Caribbean, Mixed or multiple ethnic groups) |  |
|  | 7.3 Patient sexual orientation is lesbian, gay, bisexual, queer, or asexual |  |
|  | 7.4 Patient gender identity or expression is intersex, transgender, gender non-conforming, or non-binary |  |
|  | 7.5 Patient faith is Islam, Hinduism, Sikhism, Judaism or Buddhism |  |
|  | 7.6 Patient has a known or registered disability(ies) (e.g. learning disability) |  |
| **8. Patients’ Personal Relationships** | 8.1 Patients’ Personal Relationships ANY |  |
|  | 8.2 Dissonance in relationships |  |
|  | 8.3 Poor communication in relationships |  |
| **9. Future Planning** | 9.1 Advanced Care Plan (ACP) discussed or updated |  |
|  | 9.2 RESPeCT form discussed or updated |  |
|  | 9.3 DNACPR form discussed or updated |  |
|  | 9.4 Electronic record summary completed (e.g. KIS or EPaCCS) |  |
|  | 9.5 Future planning support based on patient action plan (e.g. home modifications post-discharge) |  |
|  | 9.6 Other information support (e.g. written materials provided to complement planning conversations such as information leaflets) |  |
|  | 9.7 If YES to 9.1-5 has this been documented in discharge letter/summary |  |
|  | 9.8 Communication with or action plan for out of hours services |  |
|  | 9.9 Follow up planning with Primary Care ANY (e.g. repeat bloods in 2 weeks) |  |
|  | 9.10 Follow up planning or communication with district nursing team |  |
|  | 9.11 Follow up planning or communication with other (e.g. physio) |  |
| **10. Changing / dynamic need** | 10.1 Changing / dynamic need ANY |  |
|  | 10.2 End of life care |  |
|  | 10.2 Short prognosis |  |
|  | 10.3 Change in condition |  |
| **11. Family /Carer Support Need** | 11.1 Family / carer need ANY |  |
|  | 11.2 Emotional, psychological, spiritual need of carer |  |
|  | 11.3 Other need of carer |  |
| **12. Reason for discharge** | 12.1 Patient received respite care only |  |
|  | 12.2 Patient wishes to die / for care at home |  |
|  | 12.3 Patient wishes to die or be cared for in another place |  |
|  | 12.4 Family/carer expressed wish for patient to die /be cared for at home |  |
|  | 12.5 Family/carer wish for patient to die or be cared for in another place |  |
|  | 12.6 Patient would be better managed in community/Primary care setting |  |
|  | 12.7 Patient admitted for symptom control which has been managed/resolved |  |
|  | 12.8 Referral inappropriate/unnecessary |  |
|  | 12.9 Other reason |  |
| **13. Discharge communication** | 13.1 Patient/carer provided with verbal information about discharge |  |
|  | 13.2 Copy of discharge letter given to patient |  |
|  | 13.3 Copy of discharge letter given to carer |  |
|  | 13.4 Personalised/patient-directed discharge letter given to patient/carer (letter written *to* rather than *about* patient and contains lay explanations for jargon) |  |
|  | 13.5 Discharge letter sent by hospice to patient’s GP |  |
|  | 13.6 Patient/carer asked to deliver discharge letter to patient’s GP |  |
|  | 13.7 Patient given other written documents (e.g., leaflets on their condition) |  |
| **14. Discharge letter timing (time from patient discharge to sending discharge letter to GP)** | 14.1 Discharge letter sent on day of discharge |  |
|  | 14.2 Discharge letter sent within 48 hours of patient discharge |  |
|  | 14.3 Discharge letter sent >48 hours after patient discharge but <1 week |  |
|  | 14.4 Discharge letter sent >1 week after patient discharge but < 2 weeks |  |
|  | 14.5 Discharge letter sent >2 weeks after patient discharge but < 3 weeks |  |
|  | 14.6 Discharge letter sent more than 3 weeks after patient discharge |  |
|  | 14.7 Timing of discharge letter to GP not detailed in patient record |  |
| **15. Discharge letter timing (time from patient discharge to sending discharge letter to patient/carer)** | 15.1 Discharge letter sent/given on day of discharge |  |
|  | 15.2 Discharge letter sent/given within 48 hours of patient discharge |  |
|  | 15.3 Discharge letter sent/given > 48 hours after discharge but <1 week |  |
|  | 15.4 Discharge letter sent/given >1 week after patient discharge but < 2 weeks |  |
|  | 15.5 Discharge letter sent/given > 2 weeks after discharge but < 3 weeks |  |
|  | 15.6 Discharge letter sent/given more than 3 weeks after patient discharge |  |
|  | 15.7 Timing of discharge letter to patient/carer not detailed in patient record |  |
| **16. Non-specialist or supportive services** | 16.1 Referral made to non-specialist services |  |
|  | 16.2 Reference in discharge letter to non-specialist or supportive services |  |

**This form has been adapted and based on the “documented markers of complexity” used by Finucane et al. [supplementary material 1]*

Finucane, A.M., Swenson, C., MacArtney, J.I., et al. (2021). “What makes palliative care needs “complex”? A multisite sequential explanatory mixed methods study of patients referred for specialist palliative care” BMC palliative care. Vol. 20(1) doi: 10.1186/s12904-020-00700-3
