# Supplementary material for: Communication of palliative care needs in discharge letters from hospice providers to primary care: a multisite sequential explanatory mixed methods study
Source: BMC Palliat Care. 2022 Sep 6;21:155. doi: 10.1186/s12904-022-01038-8 (PMC9444706; doi:10.1186/s12904-022-01038-8)
Supplement: Supplementary file 2 — Additional file 2. Focus group guide. [file 12904_2022_1038_MOESM2_ESM.docx]

**Additional file 2 Focus group guide**

**Clinician focus group (part 2) guide for local collaborator**

This guide has been adapted and based on the “focus group guide” used by Finucane et al. [supplementary file 2].

Finucane, A.M., Swenson, C., MacArtney, J.I., et al. (2021). “What makes palliative care needs “complex”? A multisite sequential explanatory mixed methods study of patients referred for specialist palliative care” BMC palliative care. Vol. 20(1) doi: 10.1186/s12904-020-00700-3

*Before the focus group begins:*

- Welcome, introductions & any housekeeping points
- Local collaborator to introduce their role in the focus group – to guide discussion within the group and to aid by providing prompts and answering any questions.
- Ensure that 3-8 health professionals, clinicians and allied health professionals that work in specialist palliative care are in attendance.
- Check the participants have read and understood the participant information leaflet.
- Give them the opportunity to ask questions.
- Remind participants that taking part is entirely voluntarily and attendees can choose to listen only, stop, or leave at any time without providing a reason.
- Confirm verbally that the focus group will be recorded and active participation in the focus group indicates consent to this recording and for their data being used as outlined in the participant information leaflet.
- Write this message in the Teams chat, *“This focus group is being recorded and by taking part in this focus group you are consenting to this recording and for the recording data to be used as outlined in the participant information leaflet you were provided in advance. You may choose not to participate or to leave if you do not consent to being recorded or use of your data.”*
- Please inform participants that the study research fellow Katharine Weetman is observing the focus group (if applicable) and will be taking deidentified notes to help remember what has been said. Katharine is also available to answer any questions the group may have.
- Local collaborator to remind participants that they should feel free to share opinions and thoughts, that there are no right or wrong answers, and that the aim of the focus group is to hear the views and thoughts of participants.

**Focus group to begin**

Local collaborator to say:

*“We will now begin recording the focus group, if you do not consent to being recorded, please leave the Teams meeting now. Please avoid identifying patients, families, or staff members during the discussion. I will begin by presenting some of the data and findings from part 1 of the study which involved discharge letter review. We will then begin the focus group discussion.”*

**Data presentation**

Local collaborator run through key data points on PowerPoint from part 1 discharge letter review (10 mins)

**Discussion guide**

**Questions for local collaborator to ask the focus group:**

- What do you think are the main reasons for discharge from specialist palliative care services?
  - *Does the data presented reflect this?*
  - *What things does the data presented not capture?*
- Do reasons for discharge and complex needs differ between patients with a cancer or a non-cancer diagnosis?
- Discharge letter forms and templates may vary, what impact do you think this has on discharge communications?
  - *How do discharge letters vary?*
  - *In your opinion, what are the essential and optional elements of discharge letters from specialist to primary palliative care?*
- Why might some patients be better managed by Primary Care palliative services?
- In your experience, how well do discharge letters communicate the key issues and complex needs for a patient?
  - *How do the issues you identify on acceptance of the referral compare with the discharge issues (if any)?*
  - *How could discharge communication to primary palliative care be improved?*

**Potential themes to explore further:**

- What constitutes complex pain?
- What constitutes an advanced care plan?
- Complex needs communicated in discharge letters
- Discharge letter templates for specialist palliative/ hospice care
- Participants’ experiences of discharge communication

**Possible prompts:**

- What constitutes complex needs?
- How are palliative care needs communicated at discharge and in discharge letters?
- Is there anything you have experienced whilst providing hospice care and discharging patients to community palliative care that is important to consider when designing process and services in the future?
- How could guidance on discharge letters for specialist palliative care be improved?
- Is there anything else you feel is important that you would like to share?

*Post focus group*

- Inform participants that the focus group is now finished & stop recording
- Ask them how they found taking part & invite them to ask further questions
- Briefly remind participants what the focus group will contribute to
- Thank them for their time and state that the focus group has now finished, and participants may now leave the Teams meeting.
