# Supplementary material for: Communication of palliative care needs in discharge letters from hospice providers to primary care: a multisite sequential explanatory mixed methods study
Source: BMC Palliat Care. 2022 Sep 6;21:155. doi: 10.1186/s12904-022-01038-8 (PMC9444706; doi:10.1186/s12904-022-01038-8)
Supplement: Supplementary file 3 — Additional file 3. GRAMMS checklist. [file 12904_2022_1038_MOESM3_ESM.docx]

**Additional file 3 GRAMMS checklist**

**Reporting Guidelines**

**Good Reporting of A Mixed Methods Study” (GRAMMS)^1^**

| **Item** | **Location in manuscript** |
| --- | --- |
| 1. Describe the justification for using a mixed methods approach to the research question | Page 5, Methods - Design |
| 1. Describe the design in terms of the purpose, priority and sequence of methods | Page 5 Methods |
| 1. Describe each method in terms of sampling, data collection and analysis | This information for both the retrospective case note review and focus groups is contained in the methods section, pages 5-7 |
| 1. Describe where integration has occurred, how it has occurred and who has participated in it | This is covered in the “Triangulation of findings” section on page 7 |
| 1. Describe any limitation of one method associated with the present of the other method | Page 5 |
| 1. Describe any insights gained from mixing or integrating methods | Page 5-7 and 16-17 |

^1^ O’Cathain A, Murphy E, Nicholl J. The quality of mixed methods studies in health services research. J Health Serv Res Policy. 2008 Apr;13(2):92–8
