# Supplementary material for: Communication of palliative care needs in discharge letters from hospice providers to primary care: a multisite sequential explanatory mixed methods study
Source: BMC Palliat Care. 2022 Sep 6;21:155. doi: 10.1186/s12904-022-01038-8 (PMC9444706; doi:10.1186/s12904-022-01038-8)
Supplement: Supplementary file 4 — Additional file 4. Aggregated special category data. [file 12904_2022_1038_MOESM4_ESM.docx]

**Additional file 4 Aggregated special category data**

**Aggregated special category data**

| **Demographic** | **Variant** | **All hospices (n=250)** | |
| --- | --- | --- | --- |
|  |  | **count** | **%** |
| **Age** | 18-29 | 3 | 1.20% |
|  | 30-39 | 7 | 2.80% |
|  | 40-49 | 15 | 6.00% |
|  | 50-59 | 46 | 18.40% |
|  | 60-69 | 58 | 23.20% |
|  | 70-79 | 73 | 29.20% |
|  | 80-89 | 43 | 17.20% |
|  | 90-99 | 4 | 1.60% |
|  | 100+ | 1 | 0.40% |
|  |  | **TOTAL** | |
|  |  | 250 | 100.00% |
|  |  |  |  |
| **Gender** | Male | 135 | 54.00% |
|  | Female | 115 | 46.00% |
|  | Non-binary | 0 | 0.00% |
|  | Other | 0 | 0.00% |
|  |  | **TOTAL** | |
|  |  | 250 | 100.00% |
|  |  |  |  |
| **Ethnicity** | White | 226 | 90.40% |
|  | Mixed or multiple ethnic groups | 10 | 4.00% |
|  | Asian or Asian British | 6 | 2.40% |
|  | Black, African, Caribbean, or Black British | 1 | 0.40% |
|  | Other | 5 | 2.00% |
|  | Not documented | 2 | 0.80% |
|  |  | **TOTAL** | |
|  |  | 250 | 100.00% |
