# Supplementary material for: Communication of palliative care needs in discharge letters from hospice providers to primary care: a multisite sequential explanatory mixed methods study
Source: BMC Palliat Care. 2022 Sep 6;21:155. doi: 10.1186/s12904-022-01038-8 (PMC9444706; doi:10.1186/s12904-022-01038-8)
Supplement: Supplementary file 5 — Additional file 5. Corpus linguistics outputs. [file 12904_2022_1038_MOESM5_ESM.docx]

**Additional file 5 Corpus linguistics outputs**

*Top 100 lemmatised content words in hospice discharge letter corpus*

| **Rank** | **No. of hits** | **Content word** | **Rank** | **Hits** | **Keyword** | **Rank** | **Hits** | **Keyword** | **Rank** | **Hits** | **Keyword** |
| --- | --- | --- | --- | --- | --- | --- | --- | --- | --- | --- | --- |
| **1** | 970 | patient | **26** | 222 | need | **51** | 147 | package | **76** | 108 | infection |
| **2** | 857 | care | **27** | 208 | require | **52** | 146 | mobility | **77** | 107 | investigation |
| **3** | 774 | home | **28** | 205 | admit | **53** | 145 | improve | **78** | 107 | issue |
| **4** | 712 | pain | **29** | 200 | control | **54** | 143 | include | **79** | 105 | try |
| **5** | 688 | admission | **30** | 197 | far | **55** | 143 | nausea | **80** | 104 | effect |
| **6** | 546 | place | **31** | 196 | manage | **56** | 139 | marie | **81** | 104 | leave |
| **7** | 534 | hospice | **32** | 190 | future | **57** | 138 | curie | **82** | 103 | become |
| **8** | 508 | discharge | **33** | 190 | resuscitation | **58** | 138 | discuss | **83** | 103 | function |
| **9** | 451 | prefer | **34** | 182 | well | **59** | 137 | result | **84** | 103 | long |
| **10** | 421 | during | **35** | 179 | palliative | **60** | 134 | give | **85** | 101 | bed |
| **11** | 383 | plan | **36** | 179 | support | **61** | 133 | change | **86** | 99 | likely |
| **12** | 338 | follow | **37** | 174 | life | **62** | 132 | prognosis | **87** | 98 | disease |
| **13** | 331 | day | **38** | 170 | hospital | **63** | 130 | course | **88** | 98 | prior |
| **14** | 274 | problem | **39** | 167 | aware | **64** | 130 | management | **89** | 97 | antibiotic |
| **15** | 274 | symptom | **40** | 167 | find | **65** | 129 | nurse | **90** | 97 | main |
| **16** | 267 | feel | **41** | 166 | low | **66** | 128 | treatment | **91** | 96 | anxiety |
| **17** | 267 | time | **42** | 166 | oral | **67** | 127 | oxycodone | **92** | 96 | patch |
| **18** | 261 | team | **43** | 166 | week | **68** | 125 | see | **93** | 96 | stop |
| **19** | 250 | dose | **44** | 156 | continue | **69** | 122 | want | **94** | 94 | condition |
| **20** | 242 | community | **45** | 156 | take | **70** | 120 | review | **95** | 94 | transfer |
| **21** | 242 | due | **46** | 154 | start | **71** | 116 | morphine | **96** | 93 | syringe |
| **22** | 242 | use | **47** | 153 | good | **72** | 112 | constipation | **97** | 92 | blood |
| **23** | 228 | family | **48** | 153 | reduce | **73** | 110 | understand | **98** | 91 | concerned |
| **24** | 227 | increase | **49** | 152 | help | **74** | 109 | remain | **99** | 90 | able |
| **25** | 226 | death | **50** | 152 | medication | **75** | 108 | bowel | **100** | 90 | carer |

*Sample of 10 random concordance lines for “patient”*

| a package of care which has been requested. | **Patient** | also attended for a pre-arranged CT scan |
| --- | --- | --- |
| clot retention if tranexamic acid were to start. | **Patient** | also mentioned passing blood with his faeces in |
| as is currently the preferred place of care. | **Patient** | and family aware that they can be rereferred |
| about likely prognosis. Family are aware of | **patient** | condition and that PATIENT illness is terminal. |
| The district nurses will continue to support | **patient** | at home. PATIENT is fully aware of his |
| with him and his family prior to discharge. | **Patient** | aware of DIAGNOSIS but does not like to |
| chronic neck pain and peripheral neuropathy. | **Patient** | benefitted from an up-titration of his gabapentin |
| to radiotherapy treatment. As you are aware | **patient** | can't have an MRI scan which would |
| impairment: There were concerns about | **patient** | cognition on admission, as she was forgetful of |
| the point of transfer it was felt that | **patient** | could not be well enough to return home |

*Sample of 10 random concordance lines for “care”*

| be at the hospice for end of life | **care** | but he has stabilised albeit at a lower |
| --- | --- | --- |
| some strength in his legs. Plans for future | **care** | Community Palliative Care Team and |
| with medicine prompts and supervise personal | **care** | Family will be taking this role until package |
| death. However, he currently would prefer to be | **cared** | for and die at home. Follow Up Community |
| leg weakness. Anxious being alone. Ability to | **care** | for self rapidly diminishing. Management |
| from the [PLACE] for likely end-of-life | **care** | He had had a significant bleed from his |
| of advance care plans Preferred place of | **care** | - home, patient can't express, family's wishes |
| will be taking this role until package of | **care** | is met. PATIENT is Aware of terminal nature |
| incurable. Patient is currently still under the | **care** | of oncology and being considered for further |
| be made at the time with family and health | **care** | care professional. Hospital isn’t always the |

*Sample of 10 random concordance lines for “home”*

| to travel. PATIENT preferred place of care is | **home** | and his preferred place of death is undecided. |
| --- | --- | --- |
| care: Community palliative nurse follow up at | **home** | CPR discussed with patient: No – at risk of |
| is admission: - 1. Chest infection: admitted from | **home** | for treatment of chest infection. Completed a |
| visits so that suppositories can be given at | **home** | if needed. Community prescription has been |
| goldline. Discharged for end of life care at | **home** | - Package of Care in place - Hoist for transfer |
| relaxed since admission and is now ready for | **home** | Pain- has gout in his Left great toe, |
| referred Place of Care: Preferred place of care - | **home** | Preferred Place of Death: Preferred place of |
| had contributed significantly to his symptoms at | **home** | takes Mirtazapine 45mg OD which was unaltered |
| for symptom control. He was struggling at | **home** | with crampy abdominal pain and anxiety related |
| Anticipatory medicines available at | **home** | Yes, anticipatory medicines have been provided |

*Sample of 10 random concordance lines for “pain”*

| shortness of breath, reflux and epigastric | **pain** | A CT scan had been provisionally reported |
| --- | --- | --- |
| admitted from home for help with managing | **pain** | and her bowels. She was finding it very |
| sitting up from laying down exacerbates the | **pain** | as does coughing, sneezing and laughing. |
| by the community palliative care team for | **pain** | control and psychological support for low mood. |
| to increase the dose to 90mg as her | **pain** | had been worse. We have also increased her |
| have titrated the dose up to improve his | **pain** | however, at doses of 70mg Oxycodone MR |
| Patient was admitted with ongoing | **pain** | in left axilla and radiating down her arm |
| any recurrence of confusion. Abdominal | **pain** | is well controlled with morphine MR and |
| from home on DATE after struggling with | **pain** | . PATIENT felt his symptoms had escalated |
| daily. Over the next couple of days her | **pain** | was worse again and she described severe |

*Sample of 10 random concordance lines for “admission”*

| has deteriorated over the course of her | **admission** | and she is spending more time in bed. |
| --- | --- | --- |
| shoulder pain had been a problem prior to | **admission** | but this has not been an issue during |
| strong opioids for his pain relief; but on | **admission** | he began to need rapidly escalating doses of |
| was almost bedbound at the start of her | **admission** | Now she is able to sit on side |
| for a period of symptom control. On | **admission** | PATIENT has described how his pain had been |
| was recovering from a chest infection on | **admission** | She had completed 2 weeks of erythromycin |
| not helped her discomfort. Just prior to | **admission** | she had her oxycodone increased and was |
| resuscitation) She is not for re | **admission** | to hospital and it would not be appropriate |
| continue, and he would be appropriate for | **admission** | to hospital for management of a reversible |
| weakness, low mood/anxiety. During her | **admission** | PATIENT dexamethasone dose was increased |

*Sample of 10 random concordance lines for “place”*

| admission: Not for resuscitation Preferred | **place** | of Care: Preferred place of care – home |
| --- | --- | --- |
| Preferred place of care: Home Preferred | **place** | of death: Hospice Follow Up PATIENT will |
| supportive treatment and discussed and put in | **place** | a DNACPR form. Whilst a patient, PATIENT |
| was transferred to the hospice from the | **PLACE** | for symptom management of fatigue, |
| to enjoy what time she does have. Preferred | **place** | of care: HOME but accepting that this has |
| Details of advance care plans Preferred | **place** | of care - home Preferred place of death: |
| and this includes the hospice as preferred | **place** | of death. She does not want to be |
| an increased POC 2X4 has been put in | **place** | to allow PATIENT to be discharged. Plans |
| discuss her with the GI team at the | **PLACE** | who having done her endoscopy, felt that |
| patient can't express, family's wishes Preferred | **place** | of death: hospice, patient unable to express |

*Sample of 10 random concordance lines for “hospice”*

| Place of care: Home but is agreeable to | **hospice** | admission if required. Does not want transfer |
| --- | --- | --- |
| to be at home. He would consider further | **hospice** | admissions if things were to change at home. |
| on a modified release opiate while in the | **hospice** | but this was not tolerated due to side |
| PATIENT was discharged from the | **hospice** | but was quickly re-admitted a few hours |
| is aware he can come back to the | **hospice** | following an appropriate referral from the |
| He was transferred to the [PLACE] | **hospice** | for symptom control regarding his breathlessness, |
| and supported discharge home. While at the | **hospice** | he has attended [PLACE] hospital for radiotherapy |
| mucous plugging, was referred to the | **hospice** | inpatient unit for ongoing symptom control and |
| no improvement. Preferred place of death: | **hospice** | . Preferred place of care: Home. Follow Up Will |
| psychological support from the team here at the | **hospice** | . PATIENT has been reviewed and assessed by |

*Sample of 10 random concordance lines for “during”*

| support. Summary of clinical problems on/ | **during** | admission, including treatments, interventions |
| --- | --- | --- |
| a period of time, and then stopped. | **During** | admission, PATIENT was allocated a new flat |
| a very painful mouth and swollen lip | **during** | her admission, initially with evidence of thrush |
| thought to have only a short prognosis. | **During** | her time with us, she has deteriorated in |
| he has not had any further seizures | **during** | his admission. We have discussed seizure |
| Future planning: Resuscitation status | **during** | hospice admission: Not for resuscitation |
| prior to Christmas not discussed again | **during** | IP stay. - - Future planning: - - |
| the night and felt brighter and sharper | **during** | the day. She has now not required any |
| psychological distress contributing to pain. | **During** | this admission fentanyl patch was titrated |

*Sample of 10 random concordance lines for “plan”*

| care and has was able to engage with | **plan** | during his admission. Patient/carers |
| --- | --- | --- |
| call later in the week. We also discussed | **plans** | following discharge and that she has been |
| be implemented before he is discharged. | **Plans** | for future care: PATIENT will be re-referred |
| mood is reasonable at present. Discharge | **plan** | is being discharged to his new assisted- |
| tolerated due to side effects. 6. Discharge | **planning** | . PATIENT has been discharged to a care |
| up with oncologist as required Future | **planning** | : Resuscitation status during hospice |
| Referral received for mobility review. | **Plan** | to assess on IPU. Joint assessment with OT |
| No benefits from high dose steroids, | **planned** | to be reduced to a baseline of 2mg |
| on further exploration she had no active | **plans** | to carry this out and had multiple protective |
| also trialled with nil effect. Advanced care | **planning** | was discussed with the patient and her |

*Sample of 10 random concordance lines for “follow”*

| recently decided not to return to the DTU | **following** | a chest infection that has caused him to |
| --- | --- | --- |
| ultimately settled with senna on discharge, | **following** | an episode of diarrhoea, thought to be related |
| his admission here. On her return and | **following** | discussion regarding discharge she has |
| in place for six weeks and later removed | **following** | outpatient orthopaedic review. PATIENT has |
| to stop the anticoagulation altogether | **following** | this informed discussion with the doctors here |
| for future care: Community palliative nurse | **follow** | up at home. CPR discussed with patient: No |
| RESPECT Form in place and due to be | **followed** | up by oncology towards the end of the |
| treated for reversible causes in hospice. | **Follow** | Up Community Palliative Care follow up. |
| three carers a day. Palliative medicine | **follow** | up: Day Therapy Unit attendance at Marie |
| take home. PATIENT is under continued | **follow** | up from the haematology and urology teams |

*Sample of 10 random concordance lines for “day”*

| be discharged from the Hospice on the same | **day** | and so we will not be aware of |
| --- | --- | --- |
| and was spending time in bed during the | **day** | but was unclear if this was related to |
| If this doesn't clear up after a 14 | **day** | course we would recommend sending swabs for |
| had not opened his bowels for a few | **days** | . He initially declined laxatives, but then agree |
| arranged for him to attend the [PLACE] | **Day** | Hospice on a Tuesday for ongoing rehabilitation |
| radiotherapy to this area. PATIENT received a | **day** | of high dose steroids of 16mg while awaiting |
| to be overnight but have occurred during the | **day** | on occasion. These generally settle with |
| overlay mattress Care package: four times a | **day** | requested fast track appropriate. Palliative |
| the admission spent a short time on the | **day** | therapy unit which she did not particularly enjoy |
| concern that had deteriorated over recent | **days** | with difficulty mobilising independently and |

*Sample of 10 random concordance lines for “problem”*

| for discharge so that these are in place. | **Problems** | addressed: - Pain: has a distal left |
| --- | --- | --- |
| how to access help should it become a | **problems** | again. PATIENT had a once daily POC prior |
| investigations and strategies for potential | **problems** | ) Due to discharge over the weekend PATIENT |
| of breath and struggling at home. Main | **problems** | during this admission: 1. Dyspnoea: We have |
| have remained relatively stable. Her main | **problems** | is with reflux, particularly at night, and she |
| despite drainage. Summary of clinical | **problems** | on/during admission, including treatments |
| weaned down and she has had no further | **problems** | since then. We had discussions around her |
| Throughout her admission has had recurring | **problems** | with constipation and agitation secondary to |
| During this admission, she has not had any | **problems** | with this syringe driver compared to previous |
| investigations and strategies for potential | **problems** | ) PATIENT is being discharged home with a |

*Sample of 10 random concordance lines for “symptom”*

| admitted to the Hospice for a period of | **symptom** | assessment and control. On admission her main |
| --- | --- | --- |
| as an outpatient for gastrointestinal | **symptoms** | but was admitted acutely, and a CT scan |
| agreeable to further hospice admission for | **symptom** | control but stated during this admission, he |
| information: Admitted to the hospice for | **symptom** | control of pain and nausea and vomiting. |
| and fatigue) with a good improvement in | **symptoms** | following this. keen to continue with regular |
| to facilitate this) if he was to become | **symptomatic** | . However, without being able to stop the |
| of lung cancer, was admitted for | **symptom** | management. He had had a recent fall with |
| obstruction and discounted. His main | **symptoms** | on admission to the hospice were nausea, |
| poor mobility and fatigue being her main | **symptoms** | . She was also complaining of increased |
| pain. We noted in time that his GI | **symptoms** | worsened prior to needing to open his bowels |

*Sample of 10 random concordance lines for “feel”*

| has extra that she can take if she | **feels** | nauseous. She has not needed any extra here |
| --- | --- | --- |
| nausea and partly due to his difficulty with | **feeling** | up to preparing a meal. On admission he |
| He has adjusted well to hospice life. He | **feels** | the opioid switch has made a big difference |
| in case, on occasion, she does not | **feel** | she is able to manage the stairs at |
| such will take her car back as she | **feels** | using the Oramorph for shortness of breath is |
| but would not like to repeat this despite | **feeling** | constipated at times. The team apologise in |
| input. Mood: is a positive person and | **feels** | his mood is reasonable at present. Discharge |
| is sitting for long periods of time. He | **feels** | content with sitting in the chair most of |
| inpatient, he decided that as a result of | **feeling** | much better, he would prefer to be at |
| which he has been having at home. I | **feel** | he is well enough at present that these |

*Sample of 10 random concordance lines for “time”*

| discharged to sheltered housing with a four | **times** | a day package of care. PATIENT knows she |
| --- | --- | --- |
| and that he could deteriorate at any | **time** | , although he is hoping he may have some |
| After a dose he opened his bowels multiple | **times** | and so we decided to stop this again. |
| medically fit for home and keen to spend | **time** | at home with her husband. Many thanks for |
| familiar with this as has had it many | **times** | before. A course of [DRUG] was given and |
| 2. Bladder spasmodic pain. This was some | **times** | coincidental with the tenesmus pain and |
| been started on gabapentin 100mg three | **times** | daily as it was felt there was a |
| admission which was more marked at night | **time** | . During her stay with us, this has settled |
| cancer and her prognosis. PATIENT was at | **times** | found it hard to come to terms with, |
| mobile, but does spend most of his | **time** | in bed due to his weakness and fatigue. |

*Sample of 10 random concordance lines for “team”*

| date of when this will start). The React | **team** | will visit tonight, and her usual care package |
| --- | --- | --- |
| at the request of the oncology | **team** | , with [MEDICATION] as prophylaxis for any |
| he has declined further support from our | **team** | . We would be grateful if you could follow |
| fractures. We note that her oncology | **team** | had requested a DEXA which she failed to |
| she has been seeing the physiotherapy | **team** | and is mobilising well. Her blood pressure was |
| PV discharge and the district nursing | **team** | will kindly follow this up. Lives at home |
| For referral to community palliative care | **team** | if unable to attend and experiencing symptom |
| would ask the Community Palliative Care | **Team** | to review this dose and consider weaning |
| remainder of her life and our community | **team** | will continue to follow up but she could |
| up by the Community Palliative Care | **Team** | on discharge. I understand he will be sent |

*Sample of 10 random concordance lines for “dose”*

| dose is low compared with his background | **dose** | but does appear to be working however if |
| --- | --- | --- |
| higher doses. He is now tolerating a stable | **dose** | of 100mg twice daily, however we have asked |
| medication, and switching to equivalent | **doses** | orally — however PATIENT was reluctant to |
| in syringe driver to good effect but lowered | **dose** | due to constipation. Bowels now moving regularly |
| renal function and potassium whilst on high | **dose** | diuretics – re: check in 2 weeks initially. |
| injection. Following this, I expect her opiate | **dose** | will be reduced but will await the advice |
| were not effective, and so was a high | **dose** | of steroids. 3. Other issues addressed including |
| pressure. We have reduced his diuretic | **dose** | but no improvement has been seen. We have |
| daytime sleepiness and so the morning | **dose** | was reduced to 6.25mg. Constipation –had |
| was commenced on a fentanyl patch (the | **dose** | of which was titrated during the admission) She |

*Sample of 10 random concordance lines for “community”*

| or consider seeking advice from her | **community** | specialist nurse. Her family raised concerns |
| --- | --- | --- |
| Despite palliative care involvement in the | **community** | , they had not been able to get on |
| the district nurses will continue in the | **community** | . She has managed brilliantly on admission and |
| they would be supported at home in the | **community** | . They discussed that if he should deteriorate |
| Palliative Care Team. Please could the | **Community** | Palliative Care Team review PATIENT with |
| at [PLACE] Hospice- new referral sent | **Community** | Palliative Care Team – Future planning: |
| He was referred to the hospice by the | **community** | palliative care team for pain control and |
| Alarm Palliative medicine follow-up: | **Community** | Palliative Care Team – Future planning: |
| they will endeavour to support her in the | **community** | to the best of their abilities. Following |
| for the remainder of her life and our | **community** | team will continue to follow up but she |

*Sample of 10 random concordance lines for “due”*

| this admission her condition has deteriorated | **due** | to disease progression. She is now O2 |
| --- | --- | --- |
| appetite secondary to the cancer but also | **due** | to the symptoms he has, secondary to the |
| explained the reason for this being immobility | **due** | to the left sided weakness; also, the steroids |
| fast-track. 4. Pain control needed adjustment | **due** | pain in various body areas and Fentanyl patch |
| for his first supra-pubic catheter change in | **due** | course (his first change will not be done |
| a significant impact on him and his family. | **Due** | to symptoms is less able to do things |
| seizure control. Oral alternatives not possible | **due** | to non-compliance issues. Advice from registrar |
| urinary incontinence but this is thought to be | **due** | to poor mobility and time taken to get |
| deterioration leading to admission was likely | **due** | to a reversible process that wasn’t identified |
| secure transferring. Cannot walk long distances | **due** | to breathlessness. Reason for admission: This |

*Sample of 10 random concordance lines for “use”*

| home that she is no longer safe to | **use** | and has been advised that she will need |
| --- | --- | --- |
| Initially she had been very reluctant to | **use** | breakthrough medication and worried about |
| a rescue pack of PEG route antibiotics to | **use** | if PATIENT develops an infection which will |
| on exertion. She has been encouraged to | **use** | non pharmacological measures to help manage |
| therapist and physiotherapist discussing the | **use** | of breathing and distraction techniques. She |
| addressed: has found that he requires the | **use** | of nasal irrigation and cough assist more than |
| Amitriptyline. He has not felt the need to | **use** | Oramorph during his inpatient stay. We have |
| put it on, and he can manage to | **use** | the Cough Assist himself. He has been |
| oxygen therapy had any benefit so did not | **use** | this whilst an inpatient. He found no benefit |
| bathroom. 4. Skin — thinning due to steroid | **use** | with extensive bruising. Bilateral erythema |

*Sample of 10 random concordance lines for “family”*

| with PATIENT about likely prognosis. | **Family** | are aware of PATIENT condition and that |
| --- | --- | --- |
| if possible. If he does require admission the | **family** | would have a preference for admission to the |
| with wife. Supported by wife and extended | **family** | , such that they feel Package of care not |
| prompts and supervise personal care. | **Family** | will be taking this role until package of |
| possibly weeks/ short months. Patient and | **Family** | Awareness: Aware of diagnosis and terminal |
| when she might be on her own. The | **family** | are worried about her in those moments but |
| supply concerns: PATIENT and his | **family** | have raised concerns about problems with |
| was feeling a little disorientated, with his | **family** | needing to frequently re-orientate him. We |
| discharge planning there have been several | **family** | meetings to determine the best route forward. |
| and he felt better in himself, with his | **family** | being actively involved with his nursing care |

*Sample of 10 random concordance lines for “increase”*

| She had been struggling with | **increasing** | right sided chest pain and significant |
| --- | --- | --- |
| He has been discharged with an | **increase** | in his package of care. Preferred place of |
| her admission and we have slightly | **increased** | her insulin doses. 4. Treated with a course of |
| constipation. His opiates have been | **increased** | with benefit and he says the steroids, started |
| to increase further due to age, frailty, | **increased** | falls risk etc. We can follow up with |
| pregabalin she has not wanted to | **increase** | this at the moment. PATIENT is a very |
| this admission. Constipation: Laxatives | **increased** | with good effect. Mobility: Now mobilising |
| which he tolerated well. It is becoming | **increasingly** | difficult to find a suitable site for |
| symptoms. She was also complaining of | **increased** | nausea. Her medications, including pain |

*Sample of 10 random concordance lines for “death”*

| not like to talk about this. Place of | **death** | : PATIENT currently not ready to talk about |
| --- | --- | --- |
| difficult it is to predict. Preferred place of | **death** | : Undecided — we have started these |
| which is life-limiting Preferred place of | **death** | : not discussed as far as I am aware |
| not manageable. Preferred Place of | **Death** | : Hospice. PATIENT may be appropriate for |
| of care: Home Preferred place of | **death** | : Hospice. Follow Up PATIENT will be followed |
| Place of Death: Preferred place of | **death** | : hospice. Upon discharge wants to be looked |
| of care - hospice. Preferred Place of | **Death** | : Preferred place of death: hospice. Deciding |
| place of care - home. Preferred Place of | **Death** | : Preferred place of death: hospice. Deciding |
| place of care - home. Preferred place of | **death** | : hospice. Not for resuscitation. Would consider |
| care – nursing home. Preferred place of | **death** | : hospice. Not for attempted CPR (cardiopulmonary |
