# Supplementary material for: Communication of palliative care needs in discharge letters from hospice providers to primary care: a multisite sequential explanatory mixed methods study
Source: BMC Palliat Care. 2022 Sep 6;21:155. doi: 10.1186/s12904-022-01038-8 (PMC9444706; doi:10.1186/s12904-022-01038-8)
Supplement: Supplementary file 6 — Additional file 6. Data extraction results. [file 12904_2022_1038_MOESM6_ESM.docx]

**Additional file 6 Data extraction results**

**SPSS results and outputs for data extraction forms***

| **Reason for admission** | | | | | |
| --- | --- | --- | --- | --- | --- |
|  | | Frequency | Percent | Valid Percent | Cumulative Percent |
|  | elective admission for drain or blood transfusion | 3 | 1.2 | 1.2 | 1.2 |
|  | end of life care | 5 | 2.0 | 2.0 | 3.2 |
|  | rehabilitation | 3 | 1.2 | 1.2 | 4.4 |
|  | respite | 5 | 2.0 | 2.0 | 6.4 |
|  | Social crisis | 2 | .8 | .8 | 7.2 |
|  | symptom management | 232 | 92.8 | 92.8 | 100.0 |
|  | Total | 250 | 100.0 | 100.0 |  |

| **Duration of admission (days)** | | | | | |
| --- | --- | --- | --- | --- | --- |
|  | N | Minimum | Maximum | Mean | Std. Deviation |
| Duration of admission (days) | 250 | 1 | 240 | 21.92 | 21.212 |
| Valid N (listwise) | 250 |  |  |  |  |

| **Statistics** | | |
| --- | --- | --- |
| Duration of admission (days) | | |
| N | Valid | 250 |
| Median | | 17.00 |
| Range | | 239 |

| **1.1 Physical Symptoms ANY** | | | | | |
| --- | --- | --- | --- | --- | --- |
|  | | Frequency | Percent | Valid Percent | Cumulative Percent |
| Valid | No | 4 | 1.6 | 1.6 | 1.6 |
|  | Yes | 246 | 98.4 | 98.4 | 100.0 |
|  | Total | 250 | 100.0 | 100.0 |  |

| **1.2 Pain ANY** | | | | | |
| --- | --- | --- | --- | --- | --- |
|  | | Frequency | Percent | Valid Percent | Cumulative Percent |
| Valid | No | 56 | 22.4 | 22.4 | 22.4 |
|  | Yes | 194 | 77.6 | 77.6 | 100.0 |
|  | Total | 250 | 100.0 | 100.0 |  |
| **1.3 Complex pain** | | | | | |
|  | | Frequency | Percent | Valid Percent | Cumulative Percent |
| Valid | No | 176 | 70.4 | 70.4 | 70.4 |
|  | Yes | 74 | 29.6 | 29.6 | 100.0 |
|  | Total | 250 | 100.0 | 100.0 |  |

| **1.4 Syringe driver** | | | | | |
| --- | --- | --- | --- | --- | --- |
|  | | Frequency | Percent | Valid Percent | Cumulative Percent |
| Valid | No | 197 | 78.8 | 78.8 | 78.8 |
|  | Yes | 53 | 21.2 | 21.2 | 100.0 |
|  | Total | 250 | 100.0 | 100.0 |  |

| **1.5 Two or more types of analgesia or routes of administration prescribed** | | | | | |
| --- | --- | --- | --- | --- | --- |
|  | | Frequency | Percent | Valid Percent | Cumulative Percent |
| Valid | No | 101 | 40.4 | 40.4 | 40.4 |
|  | Yes | 149 | 59.6 | 59.6 | 100.0 |
|  | Total | 250 | 100.0 | 100.0 |  |

| **1.6 Pain required IPU admission** | | | | | |
| --- | --- | --- | --- | --- | --- |
|  | | Frequency | Percent | Valid Percent | Cumulative Percent |
| Valid | No | 129 | 51.6 | 51.6 | 51.6 |
|  | Yes | 121 | 48.4 | 48.4 | 100.0 |
|  | Total | 250 | 100.0 | 100.0 |  |

| **1.7 pain requiring high dose opiates** | | | | | |
| --- | --- | --- | --- | --- | --- |
|  | | Frequency | Percent | Valid Percent | Cumulative Percent |
| Valid | No | 232 | 92.8 | 92.8 | 92.8 |
|  | Yes | 18 | 7.2 | 7.2 | 100.0 |
|  | Total | 250 | 100.0 | 100.0 |  |

| **1.8 Any of the following prescribed: Methadone, Alfentanil, Clonazepam, Lidocaine plasters, Abstral, Nifedipine** | | | | | |
| --- | --- | --- | --- | --- | --- |
|  | | Frequency | Percent | Valid Percent | Cumulative Percent |
| Valid | No | 204 | 81.6 | 81.6 | 81.6 |
|  | Yes | 46 | 18.4 | 18.4 | 100.0 |
|  | Total | 250 | 100.0 | 100.0 |  |

| **1.9 Ketamine administered/prescribed** | | | | | |
| --- | --- | --- | --- | --- | --- |
|  | | Frequency | Percent | Valid Percent | Cumulative Percent |
| Valid | No | 240 | 96.0 | 96.0 | 96.0 |
|  | Yes | 10 | 4.0 | 4.0 | 100.0 |
|  | Total | 250 | 100.0 | 100.0 |  |

| **1.10 Pain without physiological explanation** | | | | | |
| --- | --- | --- | --- | --- | --- |
|  | | Frequency | Percent | Valid Percent | Cumulative Percent |
| Valid | No | 237 | 94.8 | 94.8 | 94.8 |
|  | Yes | 13 | 5.2 | 5.2 | 100.0 |
|  | Total | 250 | 100.0 | 100.0 |  |

| **1.11 Psychology referral made for pain management** | | | | | |
| --- | --- | --- | --- | --- | --- |
|  | | Frequency | Percent | Valid Percent | Cumulative Percent |
| Valid | No | 245 | 98.0 | 98.0 | 98.0 |
|  | Yes | 5 | 2.0 | 2.0 | 100.0 |
|  | Total | 250 | 100.0 | 100.0 |  |

| **1.12 Drug sensitivities or multiple drug allergies that limit options** | | | | | |
| --- | --- | --- | --- | --- | --- |
|  | | Frequency | Percent | Valid Percent | Cumulative Percent |
| Valid | No | 223 | 89.2 | 89.2 | 89.2 |
|  | Yes | 27 | 10.8 | 10.8 | 100.0 |
|  | Total | 250 | 100.0 | 100.0 |  |
| **1.13 Patient has history of /concurrent drug abuse** | | | | | |
|  | | Frequency | Percent | Valid Percent | Cumulative Percent |
| Valid | No | 246 | 98.4 | 98.4 | 98.4 |
|  | Yes | 4 | 1.6 | 1.6 | 100.0 |
|  | Total | 250 | 100.0 | 100.0 |  |

| **1.14 Family members or person in home setting has history of drug abuse** | | | | | |
| --- | --- | --- | --- | --- | --- |
|  | | Frequency | Percent | Valid Percent | Cumulative Percent |
| Valid | No | 249 | 99.6 | 99.6 | 99.6 |
|  | Yes | 1 | .4 | .4 | 100.0 |
|  | Total | 250 | 100.0 | 100.0 |  |

| **1.15 Patient requires interventional anaesthetics** | | | | | |
| --- | --- | --- | --- | --- | --- |
|  | | Frequency | Percent | Valid Percent | Cumulative Percent |
| Valid | No | 241 | 96.4 | 96.4 | 96.4 |
|  | Yes | 9 | 3.6 | 3.6 | 100.0 |
|  | Total | 250 | 100.0 | 100.0 |  |

| **1.16 Anticipatory prescribing/ just in case medicines** | | | | | |
| --- | --- | --- | --- | --- | --- |
|  | | Frequency | Percent | Valid Percent | Cumulative Percent |
| Valid | No | 141 | 56.4 | 56.4 | 56.4 |
|  | Yes | 109 | 43.6 | 43.6 | 100.0 |
|  | Total | 250 | 100.0 | 100.0 |  |

| **1.17 Breathlessness / shortness of breath/ secretions** | | | | | |
| --- | --- | --- | --- | --- | --- |
|  | | Frequency | Percent | Valid Percent | Cumulative Percent |
| Valid | No | 171 | 68.4 | 68.4 | 68.4 |
|  | Yes | 79 | 31.6 | 31.6 | 100.0 |
|  | Total | 250 | 100.0 | 100.0 |  |

| **1.18 Nausea/ vomiting** | | | | | |
| --- | --- | --- | --- | --- | --- |
|  | | Frequency | Percent | Valid Percent | Cumulative Percent |
| Valid | No | 196 | 78.4 | 78.4 | 78.4 |
|  | Yes | 54 | 21.6 | 21.6 | 100.0 |
|  | Total | 250 | 100.0 | 100.0 |  |

| **1.19 Confusion** | | | | | |
| --- | --- | --- | --- | --- | --- |
|  | | Frequency | Percent | Valid Percent | Cumulative Percent |
| Valid | No | 217 | 86.8 | 86.8 | 86.8 |
|  | Yes | 33 | 13.2 | 13.2 | 100.0 |
|  | Total | 250 | 100.0 | 100.0 |  |

| **1.20 Agitation** | | | | | |
| --- | --- | --- | --- | --- | --- |
|  | | Frequency | Percent | Valid Percent | Cumulative Percent |
| Valid | No | 240 | 96.0 | 96.0 | 96.0 |
|  | Yes | 10 | 4.0 | 4.0 | 100.0 |
|  | Total | 250 | 100.0 | 100.0 |  |

| **1.21 Constipation** | | | | | |
| --- | --- | --- | --- | --- | --- |
|  | | Frequency | Percent | Valid Percent | Cumulative Percent |
| Valid | No | 163 | 65.2 | 65.2 | 65.2 |
|  | Yes | 87 | 34.8 | 34.8 | 100.0 |
|  | Total | 250 | 100.0 | 100.0 |  |

| **1.22 Fatigue** | | | | | |
| --- | --- | --- | --- | --- | --- |
|  | | Frequency | Percent | Valid Percent | Cumulative Percent |
| Valid | No | 189 | 75.6 | 75.6 | 75.6 |
|  | Yes | 61 | 24.4 | 24.4 | 100.0 |
|  | Total | 250 | 100.0 | 100.0 |  |

| **1.23 Other** | | | | | |
| --- | --- | --- | --- | --- | --- |
|  | | Frequency | Percent | Valid Percent | Cumulative Percent |
| Valid | No | 90 | 36.0 | 36.0 | 36.0 |
|  | Yes | 160 | 64.0 | 64.0 | 100.0 |
|  | Total | 250 | 100.0 | 100.0 |  |
| **2.1 Psychological / Emotional Need (of patient) ANY** | | | | | |
|  | | Frequency | Percent | Valid Percent | Cumulative Percent |
| Valid | No | 134 | 53.6 | 53.6 | 53.6 |
|  | Yes | 116 | 46.4 | 46.4 | 100.0 |
|  | Total | 250 | 100.0 | 100.0 |  |

| **2.2 Psychological/emotional needs of patient relating to anxiety** | | | | | |
| --- | --- | --- | --- | --- | --- |
|  | | Frequency | Percent | Valid Percent | Cumulative Percent |
| Valid | No | 188 | 75.2 | 75.2 | 75.2 |
|  | Yes | 62 | 24.8 | 24.8 | 100.0 |
|  | Total | 250 | 100.0 | 100.0 |  |

| **2.3 Psychological/emotional needs of patient relating to depression** | | | | | |
| --- | --- | --- | --- | --- | --- |
|  | | Frequency | Percent | Valid Percent | Cumulative Percent |
| Valid | No | 224 | 89.6 | 89.6 | 89.6 |
|  | Yes | 26 | 10.4 | 10.4 | 100.0 |
|  | Total | 250 | 100.0 | 100.0 |  |

| **2.4 Psychological/emotional needs of patient relating to anger** | | | | | |
| --- | --- | --- | --- | --- | --- |
|  | | Frequency | Percent | Valid Percent | Cumulative Percent |
| Valid | No | 243 | 97.2 | 97.2 | 97.2 |
|  | Yes | 7 | 2.8 | 2.8 | 100.0 |
|  | Total | 250 | 100.0 | 100.0 |  |

| **2.5 Psychological/emotional needs of patient relating to avoidance** | | | | | |
| --- | --- | --- | --- | --- | --- |
|  | | Frequency | Percent | Valid Percent | Cumulative Percent |
| Valid | No | 249 | 99.6 | 99.6 | 99.6 |
|  | Yes | 1 | .4 | .4 | 100.0 |
|  | Total | 250 | 100.0 | 100.0 |  |

| **2.6 Psychological/emotional needs of patient relating to collusion** | | | | | |
| --- | --- | --- | --- | --- | --- |
|  | | Frequency | Percent | Valid Percent | Cumulative Percent |
| Valid | No | 250 | 100.0 | 100.0 | 100.0 |

| **2.7 Psychological/emotional needs of patient specifically relating to grief** | | | | | |
| --- | --- | --- | --- | --- | --- |
|  | | Frequency | Percent | Valid Percent | Cumulative Percent |
| Valid | No | 242 | 96.8 | 96.8 | 96.8 |
|  | Yes | 8 | 3.2 | 3.2 | 100.0 |
|  | Total | 250 | 100.0 | 100.0 |  |
| **2.8 Psychological/emotional needs of carer specifically relating to grief** | | | | | |
|  | | Frequency | Percent | Valid Percent | Cumulative Percent |
| Valid | No | 249 | 99.6 | 99.6 | 99.6 |
|  | Yes | 1 | .4 | .4 | 100.0 |
|  | Total | 250 | 100.0 | 100.0 |  |

| **2.9 Psychological/emotional needs of family/friends specifically relating to grief** | | | | | |
| --- | --- | --- | --- | --- | --- |
|  | | Frequency | Percent | Valid Percent | Cumulative Percent |
| Valid | No | 249 | 99.6 | 99.6 | 99.6 |
|  | Yes | 1 | .4 | .4 | 100.0 |
|  | Total | 250 | 100.0 | 100.0 |  |
| **2.10 Psychological/ Emotional Need of carer ANY (if relevant)** | | | | | |
|  | | Frequency | Percent | Valid Percent | Cumulative Percent |
| Valid | No | 236 | 94.4 | 94.4 | 94.4 |
|  | Yes | 14 | 5.6 | 5.6 | 100.0 |
|  | Total | 250 | 100.0 | 100.0 |  |

| **2.11 Psychological/ Emotional Need of family/friends ANY (e.g., depression)** | | | | | |
| --- | --- | --- | --- | --- | --- |
|  | | Frequency | Percent | Valid Percent | Cumulative Percent |
| Valid | No | 238 | 95.2 | 95.2 | 95.2 |
|  | Yes | 12 | 4.8 | 4.8 | 100.0 |
|  | Total | 250 | 100.0 | 100.0 |  |

| **2.12 Spiritual needs of patient** | | | | | |
| --- | --- | --- | --- | --- | --- |
|  | | Frequency | Percent | Valid Percent | Cumulative Percent |
| Valid | No | 244 | 97.6 | 97.6 | 97.6 |
|  | Yes | 6 | 2.4 | 2.4 | 100.0 |
|  | Total | 250 | 100.0 | 100.0 |  |

| **2.13 Spiritual needs of carer (if relevant)** | | | | | |
| --- | --- | --- | --- | --- | --- |
|  | | Frequency | Percent | Valid Percent | Cumulative Percent |
| Valid | No | 250 | 100.0 | 100.0 | 100.0 |

| **2.14 Spiritual needs of family/friends** | | | | | |
| --- | --- | --- | --- | --- | --- |
|  | | Frequency | Percent | Valid Percent | Cumulative Percent |
| Valid | No | 249 | 99.6 | 99.6 | 99.6 |
|  | Yes | 1 | .4 | .4 | 100.0 |
|  | Total | 250 | 100.0 | 100.0 |  |

| **3.1 Active co-morbidities ANY** | | | | | |
| --- | --- | --- | --- | --- | --- |
|  | | Frequency | Percent | Valid Percent | Cumulative Percent |
| Valid | No | 136 | 54.4 | 54.4 | 54.4 |
|  | Yes-1 | 48 | 19.2 | 19.2 | 73.6 |
|  | Yes-2 | 25 | 10.0 | 10.0 | 83.6 |
|  | Yes-3 | 19 | 7.6 | 7.6 | 91.2 |
|  | Yes-4 | 11 | 4.4 | 4.4 | 95.6 |
|  | Yes-5 | 6 | 2.4 | 2.4 | 98.0 |
|  | Yes-6 | 3 | 1.2 | 1.2 | 99.2 |
|  | Yes-7 | 1 | .4 | .4 | 99.6 |
|  | Yes-8 | 1 | .4 | .4 | 100.0 |
|  | Total | 250 | 100.0 | 100.0 |  |

| **3.2 Patient diagnosed with cancer** | | | | | |
| --- | --- | --- | --- | --- | --- |
|  | | Frequency | Percent | Valid Percent | Cumulative Percent |
| Valid | No | 45 | 18.0 | 18.0 | 18.0 |
|  | Yes | 205 | 82.0 | 82.0 | 100.0 |
|  | Total | 250 | 100.0 | 100.0 |  |

| **3.3 Cancer is primary diagnosis** | | | | | |
| --- | --- | --- | --- | --- | --- |
|  | | Frequency | Percent | Valid Percent | Cumulative Percent |
| Valid | No | 51 | 20.4 | 20.4 | 20.4 |
|  | Yes | 199 | 79.6 | 79.6 | 100.0 |
|  | Total | 250 | 100.0 | 100.0 |  |

| **4.1 Functional care needs ANY** | | | | | |
| --- | --- | --- | --- | --- | --- |
|  | | Frequency | Percent | Valid Percent | Cumulative Percent |
| Valid | No | 126 | 50.4 | 50.4 | 50.4 |
|  | Yes | 124 | 49.6 | 49.6 | 100.0 |
|  | Total | 250 | 100.0 | 100.0 |  |

| **5.1 Social situation (needs) ANY** | | | | | |
| --- | --- | --- | --- | --- | --- |
|  | | Frequency | Percent | Valid Percent | Cumulative Percent |
| Valid | No | 161 | 64.4 | 64.4 | 64.4 |
|  | Yes | 89 | 35.6 | 35.6 | 100.0 |
|  | Total | 250 | 100.0 | 100.0 |  |

| **5.2 Social exclusion or isolation** | | | | | |
| --- | --- | --- | --- | --- | --- |
|  | | Frequency | Percent | Valid Percent | Cumulative Percent |
| Valid | No | 239 | 95.6 | 95.6 | 95.6 |
|  | Yes | 11 | 4.4 | 4.4 | 100.0 |
|  | Total | 250 | 100.0 | 100.0 |  |
| **5.3 Social responsibilities** | | | | | |
|  | | Frequency | Percent | Valid Percent | Cumulative Percent |
| Valid | No | 248 | 99.2 | 99.2 | 99.2 |
|  | Yes | 2 | .8 | .8 | 100.0 |
|  | Total | 250 | 100.0 | 100.0 |  |

| **5.4 Housing** | | | | | |
| --- | --- | --- | --- | --- | --- |
|  | | Frequency | Percent | Valid Percent | Cumulative Percent |
| Valid | No | 230 | 92.0 | 92.0 | 92.0 |
|  | Yes | 20 | 8.0 | 8.0 | 100.0 |
|  | Total | 250 | 100.0 | 100.0 |  |

| **5.5 Other social factor** | | | | | |
| --- | --- | --- | --- | --- | --- |
|  | | Frequency | Percent | Valid Percent | Cumulative Percent |
| Valid | No | 225 | 90.0 | 90.0 | 90.0 |
|  | Yes | 25 | 10.0 | 10.0 | 100.0 |
|  | Total | 250 | 100.0 | 100.0 |  |

| **6.1 Capacity / Communication needs ANY** | | | | | |
| --- | --- | --- | --- | --- | --- |
|  | | Frequency | Percent | Valid Percent | Cumulative Percent |
| Valid | No | 223 | 89.2 | 89.2 | 89.2 |
|  | Yes | 27 | 10.8 | 10.8 | 100.0 |
|  | Total | 250 | 100.0 | 100.0 |  |

| **6.2 Capacity needs** | | | | | |
| --- | --- | --- | --- | --- | --- |
|  | | Frequency | Percent | Valid Percent | Cumulative Percent |
| Valid | No | 245 | 98.0 | 98.0 | 98.0 |
|  | Yes | 5 | 2.0 | 2.0 | 100.0 |
|  | Total | 250 | 100.0 | 100.0 |  |

| **6.3 Communication needs (e.g., deaf/hearing loss)** | | | | | |
| --- | --- | --- | --- | --- | --- |
|  | | Frequency | Percent | Valid Percent | Cumulative Percent |
| Valid | No | 234 | 93.6 | 93.6 | 93.6 |
|  | Yes | 16 | 6.4 | 6.4 | 100.0 |
|  | Total | 250 | 100.0 | 100.0 |  |

| **7.1 Patients coping mechanisms/behaviours ANY** | | | | | |
| --- | --- | --- | --- | --- | --- |
|  | | Frequency | Percent | Valid Percent | Cumulative Percent |
| Valid | No | 227 | 90.8 | 90.8 | 90.8 |
|  | Yes | 23 | 9.2 | 9.2 | 100.0 |
|  | Total | 250 | 100.0 | 100.0 |  |

| **7.2 Patient ethnicity or race belonging to ethnic minority group (Asian, Black, African, Caribbean, Mixed or multiple ethnic groups)** | | | | | |
| --- | --- | --- | --- | --- | --- |
|  | | Frequency | Percent | Valid Percent | Cumulative Percent |
| Valid | No | 243 | 97.2 | 97.2 | 97.2 |
|  | Yes | 7 | 2.8 | 2.8 | 100.0 |
|  | Total | 250 | 100.0 | 100.0 |  |

| **7.3 Patient sexual orientation is lesbian, gay, bisexual, queer, or asexual** | | | | | |
| --- | --- | --- | --- | --- | --- |
|  | | Frequency | Percent | Valid Percent | Cumulative Percent |
| Valid | No | 249 | 99.6 | 99.6 | 99.6 |
|  | Yes | 1 | .4 | .4 | 100.0 |
|  | Total | 250 | 100.0 | 100.0 |  |

| **7.4 Patient gender identity or expression is intersex, transgender, gender non-conforming, or non-binary** | | | | | |
| --- | --- | --- | --- | --- | --- |
|  | | Frequency | Percent | Valid Percent | Cumulative Percent |
| Valid | No | 250 | 100.0 | 100.0 | 100.0 |

| **7.5 Patient faith is Islam, Hinduism, Sikhism, Judaism or Buddhism** | | | | | |
| --- | --- | --- | --- | --- | --- |
|  | | Frequency | Percent | Valid Percent | Cumulative Percent |
| Valid | No | 248 | 99.2 | 99.2 | 99.2 |
|  | Yes | 2 | .8 | .8 | 100.0 |
|  | Total | 250 | 100.0 | 100.0 |  |

| **7.6 Patient has a known or registered disability(ies)** | | | | | |
| --- | --- | --- | --- | --- | --- |
|  | | Frequency | Percent | Valid Percent | Cumulative Percent |
| Valid | No | 249 | 99.6 | 99.6 | 99.6 |
|  | Yes | 1 | .4 | .4 | 100.0 |
|  | Total | 250 | 100.0 | 100.0 |  |
| **8.1 Patients’ Personal Relationships ANY** | | | | | |
|  | | Frequency | Percent | Valid Percent | Cumulative Percent |
| Valid | No | 174 | 69.6 | 69.6 | 69.6 |
|  | Yes | 76 | 30.4 | 30.4 | 100.0 |
|  | Total | 250 | 100.0 | 100.0 |  |

| **8.2 Dissonance in relationships** | | | | | |
| --- | --- | --- | --- | --- | --- |
|  | | Frequency | Percent | Valid Percent | Cumulative Percent |
| Valid | No | 247 | 98.8 | 98.8 | 98.8 |
|  | Yes | 3 | 1.2 | 1.2 | 100.0 |
|  | Total | 250 | 100.0 | 100.0 |  |

| **8.3 Poor communication in relationships** | | | | | |
| --- | --- | --- | --- | --- | --- |
|  | | Frequency | Percent | Valid Percent | Cumulative Percent |
| Valid | No | 247 | 98.8 | 98.8 | 98.8 |
|  | Yes | 3 | 1.2 | 1.2 | 100.0 |
|  | Total | 250 | 100.0 | 100.0 |  |

| **9.1 Advanced Care Plan (ACP) discussed or updated** | | | | | |
| --- | --- | --- | --- | --- | --- |
|  | | Frequency | Percent | Valid Percent | Cumulative Percent |
| Valid | No | 28 | 11.2 | 11.2 | 11.2 |
|  | Yes | 222 | 88.8 | 88.8 | 100.0 |
|  | Total | 250 | 100.0 | 100.0 |  |

| **9.2 ReSPECT form discussed or updated** | | | | | |
| --- | --- | --- | --- | --- | --- |
|  | | Frequency | Percent | Valid Percent | Cumulative Percent |
| Valid | No | 201 | 80.4 | 80.4 | 80.4 |
|  | Yes | 49 | 19.6 | 19.6 | 100.0 |
|  | Total | 250 | 100.0 | 100.0 |  |

| **9.3 DNACPR form discussed or updated** | | | | | |
| --- | --- | --- | --- | --- | --- |
|  | | Frequency | Percent | Valid Percent | Cumulative Percent |
| Valid | No | 58 | 23.2 | 23.2 | 23.2 |
|  | Yes | 192 | 76.8 | 76.8 | 100.0 |
|  | Total | 250 | 100.0 | 100.0 |  |

| **9.4 Electronic record summary completed (e.g. (e)KIS)** | | | | | |
| --- | --- | --- | --- | --- | --- |
|  | | Frequency | Percent | Valid Percent | Cumulative Percent |
| Valid | No | 250 | 100.0 | 100.0 | 100.0 |
| **9.5 Future planning support based on patient action plan (e.g., home modifications post-discharge)** | | | | | |
|  | | Frequency | Percent | Valid Percent | Cumulative Percent |
| Valid | No | 164 | 65.6 | 65.6 | 65.6 |
|  | Yes | 86 | 34.4 | 34.4 | 100.0 |
|  | Total | 250 | 100.0 | 100.0 |  |

| **9.6 Other information support** | | | | | |
| --- | --- | --- | --- | --- | --- |
|  | | Frequency | Percent | Valid Percent | Cumulative Percent |
| Valid | No | 247 | 98.8 | 98.8 | 98.8 |
|  | Yes | 3 | 1.2 | 1.2 | 100.0 |
|  | Total | 250 | 100.0 | 100.0 |  |

| **9.7 If YES to 9.1-5 has this been documented in discharge letter/summary** | | | | | |
| --- | --- | --- | --- | --- | --- |
|  | | Frequency | Percent | Valid Percent | Cumulative Percent |
| Valid | No | 82 | 32.8 | 32.8 | 32.8 |
|  | Yes | 168 | 67.2 | 67.2 | 100.0 |
|  | Total | 250 | 100.0 | 100.0 |  |
| **9.8 Communication with or action plan for out of hours services** | | | | | |
|  | | Frequency | Percent | Valid Percent | Cumulative Percent |
| Valid | No | 154 | 61.6 | 61.6 | 61.6 |
|  | Yes | 96 | 38.4 | 38.4 | 100.0 |
|  | Total | 250 | 100.0 | 100.0 |  |

| **9.9 Follow up planning with Primary Care ANY (e.g., repeat bloods in 2 weeks)** | | | | | |
| --- | --- | --- | --- | --- | --- |
|  | | Frequency | Percent | Valid Percent | Cumulative Percent |
| Valid | No | 208 | 83.2 | 83.2 | 83.2 |
|  | Yes | 42 | 16.8 | 16.8 | 100.0 |
|  | Total | 250 | 100.0 | 100.0 |  |
| **9.10 Follow up planning or communication with district nursing team** | | | | | |
|  | | Frequency | Percent | Valid Percent | Cumulative Percent |
| Valid | No | 151 | 60.4 | 60.4 | 60.4 |
|  | Yes | 99 | 39.6 | 39.6 | 100.0 |
|  | Total | 250 | 100.0 | 100.0 |  |

| **9.11 Follow up planning or communication with other (e.g., physio)** | | | | | |
| --- | --- | --- | --- | --- | --- |
|  | | Frequency | Percent | Valid Percent | Cumulative Percent |
| Valid | No | 104 | 41.6 | 41.6 | 41.6 |
|  | Yes | 146 | 58.4 | 58.4 | 100.0 |
|  | Total | 250 | 100.0 | 100.0 |  |

| \| **10.1 Changing / dynamic need ANY** \| \| \| \| \| \| \| --- \| --- \| --- \| --- \| --- \| --- \| \|  \| \| Frequency \| Percent \| Valid Percent \| Cumulative Percent \| \| Valid \| No \| 124 \| 49.6 \| 49.6 \| 49.6 \| \| Yes \| 126 \| 50.4 \| 50.4 \| 100.0 \| \| Total \| 250 \| 100.0 \| 100.0 \|  \|   **10.2 End of life care** | | | | | |
| --- | --- | --- | --- | --- | --- | --- | --- | --- | --- | --- | --- | --- | --- | --- | --- | --- | --- | --- | --- | --- | --- | --- | --- | --- | --- | --- | --- | --- | --- | --- | --- | --- | --- |
|  | | Frequency | Percent | Valid Percent | Cumulative Percent |
| Valid | No | 235 | 94.0 | 94.0 | 94.0 |
|  | Yes | 15 | 6.0 | 6.0 | 100.0 |
|  | Total | 250 | 100.0 | 100.0 |  |

| **10.3 Short prognosis** | | | | | |
| --- | --- | --- | --- | --- | --- |
|  | | Frequency | Percent | Valid Percent | Cumulative Percent |
| Valid | No | 222 | 88.8 | 88.8 | 88.8 |
|  | Yes | 28 | 11.2 | 11.2 | 100.0 |
|  | Total | 250 | 100.0 | 100.0 |  |

| **10.4 Change in condition** | | | | | |
| --- | --- | --- | --- | --- | --- |
|  | | Frequency | Percent | Valid Percent | Cumulative Percent |
| Valid | No | 164 | 65.6 | 65.6 | 65.6 |
|  | Yes | 86 | 34.4 | 34.4 | 100.0 |
|  | Total | 250 | 100.0 | 100.0 |  |

| **11.1 Family / carer need ANY** | | | | | |
| --- | --- | --- | --- | --- | --- |
|  | | Frequency | Percent | Valid Percent | Cumulative Percent |
| Valid | No | 219 | 87.6 | 87.6 | 87.6 |
|  | Yes | 31 | 12.4 | 12.4 | 100.0 |
|  | Total | 250 | 100.0 | 100.0 |  |

| **12.1 Patient received respite care only** | | | | | |
| --- | --- | --- | --- | --- | --- |
|  | | Frequency | Percent | Valid Percent | Cumulative Percent |
| Valid | No | 244 | 97.6 | 97.6 | 97.6 |
|  | Yes | 6 | 2.4 | 2.4 | 100.0 |
|  | Total | 250 | 100.0 | 100.0 |  |

| **12.2 Patient wishes to die / for care at home** | | | | | |
| --- | --- | --- | --- | --- | --- |
|  | | Frequency | Percent | Valid Percent | Cumulative Percent |
| Valid | No | 157 | 62.8 | 62.8 | 62.8 |
|  | Yes | 93 | 37.2 | 37.2 | 100.0 |
|  | Total | 250 | 100.0 | 100.0 |  |

| **12.3 Patient wishes to die or be cared for in another place** | | | | | |
| --- | --- | --- | --- | --- | --- |
|  | | Frequency | Percent | Valid Percent | Cumulative Percent |
| Valid | No | 228 | 91.2 | 91.2 | 91.2 |
|  | Yes | 22 | 8.8 | 8.8 | 100.0 |
|  | Total | 250 | 100.0 | 100.0 |  |

| **12.4 Family/carer expressed wish for patient to die /be cared for at home** | | | | | |
| --- | --- | --- | --- | --- | --- |
|  | | Frequency | Percent | Valid Percent | Cumulative Percent |
| Valid | No | 245 | 98.0 | 98.0 | 98.0 |
|  | Yes | 5 | 2.0 | 2.0 | 100.0 |
|  | Total | 250 | 100.0 | 100.0 |  |
| **12.5 Family/carer wish for patient to die or be cared for in another place** | | | | | |
|  | | Frequency | Percent | Valid Percent | Cumulative Percent |
| Valid | No | 249 | 99.6 | 99.6 | 99.6 |
|  | Yes | 1 | .4 | .4 | 100.0 |
|  | Total | 250 | 100.0 | 100.0 |  |

| **12.6 Patient would be better managed in community/Primary care setting** | | | | | |
| --- | --- | --- | --- | --- | --- |
|  | | Frequency | Percent | Valid Percent | Cumulative Percent |
| Valid | No | 240 | 96.0 | 96.0 | 96.0 |
|  | Yes | 10 | 4.0 | 4.0 | 100.0 |
|  | Total | 250 | 100.0 | 100.0 |  |

| **12.7 Patient admitted for symptom management which has been managed/resolved** | | | | | |
| --- | --- | --- | --- | --- | --- |
|  | | Frequency | Percent | Valid Percent | Cumulative Percent |
| Valid | No | 62 | 24.8 | 24.8 | 24.8 |
|  | Yes | 188 | 75.2 | 75.2 | 100.0 |
|  | Total | 250 | 100.0 | 100.0 |  |

| **12.8 Referral inappropriate/unnecessary** | | | | | |
| --- | --- | --- | --- | --- | --- |
|  | | Frequency | Percent | Valid Percent | Cumulative Percent |
| Valid | No | 249 | 99.6 | 99.6 | 99.6 |
|  | Yes | 1 | .4 | .4 | 100.0 |
|  | Total | 250 | 100.0 | 100.0 |  |

| **12.9 Other reason** | | | | | |
| --- | --- | --- | --- | --- | --- |
|  | | Frequency | Percent | Valid Percent | Cumulative Percent |
| Valid | No | 228 | 91.2 | 91.2 | 91.2 |
|  | Yes | 22 | 8.8 | 8.8 | 100.0 |
|  | Total | 250 | 100.0 | 100.0 |  |

| **13.1 Patient/carer provided with verbal information about discharge** | | | | | |
| --- | --- | --- | --- | --- | --- |
|  | | Frequency | Percent | Valid Percent | Cumulative Percent |
| Valid | No | 120 | 48.0 | 48.0 | 48.0 |
|  | Yes | 130 | 52.0 | 52.0 | 100.0 |
|  | Total | 250 | 100.0 | 100.0 |  |

| **13.2 Copy of discharge letter given to patient (recorded in letter)** | | | | | |
| --- | --- | --- | --- | --- | --- |
|  | | Frequency | Percent | Valid Percent | Cumulative Percent |
| Valid | No | 248 | 99.2 | 99.2 | 99.2 |
|  | Yes | 2 | .8 | .8 | 100.0 |
|  | Total | 250 | 100.0 | 100.0 |  |

| **13.3 Copy of discharge letter given to carer (recorded in letter)** | | | | | |
| --- | --- | --- | --- | --- | --- |
|  | | Frequency | Percent | Valid Percent | Cumulative Percent |
| Valid | No | 249 | 99.6 | 99.6 | 99.6 |
|  | Yes | 1 | .4 | .4 | 100.0 |
|  | Total | 250 | 100.0 | 100.0 |  |

| **13.4 Personalised/patient-directed discharge letter given to patient/carer** | | | | | |
| --- | --- | --- | --- | --- | --- |
|  | | Frequency | Percent | Valid Percent | Cumulative Percent |
| Valid | No | 202 | 80.8 | 80.8 | 80.8 |
|  | Yes | 48 | 19.2 | 19.2 | 100.0 |
|  | Total | 250 | 100.0 | 100.0 |  |

| **13.5 Discharge letter sent by hospice to patient’s GP (recorded in discharge letter)** | | | | | |
| --- | --- | --- | --- | --- | --- |
|  | | Frequency | Percent | Valid Percent | Cumulative Percent |
| Valid | No | 50 | 20.0 | 20.0 | 20.0 |
|  | Yes | 200 | 80.0 | 80.0 | 100.0 |
|  | Total | 250 | 100.0 | 100.0 |  |
| **13.6 Patient/carer asked to deliver discharge letter to patient’s GP** | | | | | |
|  | | Frequency | Percent | Valid Percent | Cumulative Percent |
| Valid | No | 244 | 97.6 | 97.6 | 97.6 |
|  | Yes | 6 | 2.4 | 2.4 | 100.0 |
|  | Total | 250 | 100.0 | 100.0 |  |

| **13.7 Patient given other written documents (e.g., leaflets on their condition)** | | | | | |
| --- | --- | --- | --- | --- | --- |
|  | | Frequency | Percent | Valid Percent | Cumulative Percent |
| Valid | No | 249 | 99.6 | 99.6 | 99.6 |
|  | Yes | 1 | .4 | .4 | 100.0 |
|  | Total | 250 | 100.0 | 100.0 |  |

**14.1-7 Timing of when discharge letter sent to GP [in patient record]**

|  | | Frequency | Percent | Cumulative Percent | |
| --- | --- | --- | --- | --- | --- |
|  | On the day of discharge | 113 | 45.2 | 45.2 | |
|  | <48 hours of discharge | 45 | 18.0 | 63.2 | |
|  | >48 hours after patient discharge but <1 week | 35 | 14.0 | 77.2 | |
|  | >1 week after patient discharge but <2 weeks | 6 | 2.4 | 79.6 | |
|  | >2 weeks after patient discharge but <3 weeks | 2 | .8 | 80.4 | |
|  | More than 3 weeks after discharge | 0 | 0 | 80.4 | |
|  | Not recorded | 49 | 19.6 | 100 | |
|  | Total | 250 | 100.0 | 100.0 | |
| **15.1-7 Timing of when discharge letter sent /given to patient/carer [according to patient record]** | | | | |  |
|  | | Frequency | Percent | Cumulative Percent | |
|  | On the day of discharge | 53 | 21.2 | 21.2 | |
|  | <48 hours of discharge | 0 | 0 | 21.2 | |
|  | >48 hours after patient discharge but <1 week | 0 | 0 | 21.2 | |
|  | >1 week after patient discharge but <2 weeks | 0 | 0 | 21.2 | |
|  | >2 weeks after patient discharge but <3 weeks | 0 | 0 | 21.2 | |
|  | More than 3 weeks after discharge | 0 | 0 | 21.2 | |
|  | Not recorded (including N/A) | 197 | 78.8 | 100 | |
|  | Total | 250 | 100.0 | 100.0 | |

| **16.1 Referral made to non-specialist services** | | | | | |
| --- | --- | --- | --- | --- | --- |
|  | | Frequency | Percent | Valid Percent | Cumulative Percent |
| Valid | No | 199 | 79.6 | 79.6 | 79.6 |
|  | Yes | 51 | 20.4 | 20.4 | 100.0 |
|  | Total | 250 | 100.0 | 100.0 |  |

| **16.2 Reference in discharge letter to non-specialist or supportive services** | | | | | |
| --- | --- | --- | --- | --- | --- |
|  | | Frequency | Percent | Valid Percent | Cumulative Percent |
| Valid | No | 195 | 78.0 | 78.0 | 78.0 |
|  | Yes | 55 | 22.0 | 22.0 | 100.0 |
|  | Total | 250 | 100.0 | 100.0 |  |

**For most sections, extractors were not limited to a single answer (see supplementary file 1)*
